# Genome-Wide Analyses of the Genetic Screening of C_2_H_2_-Type Zinc Finger Transcription Factors and Abiotic and Biotic Stress Responses in Tomato (*Solanum lycopersicum*) Based on RNA-Seq Data

**DOI:** 10.3389/fgene.2020.00540

**Published:** 2020-05-28

**Authors:** Tingting Zhao, Tairu Wu, Jia Zhang, Ziyu Wang, Tong Pei, Huanhuan Yang, Jingfu Li, Xiangyang Xu

**Affiliations:** ^1^Laboratory of Genetic Breeding in Tomato, College of Horticulture and Landscape Architecture, Northeast Agricultural University, Harbin, China; ^2^Key Laboratory of Biology and Genetic Improvement of Horticultural Crops (Northeast Region), Ministry of Agriculture and Rural Affairs, Northeast Agricultural University, Harbin, China

**Keywords:** C_2_H_2_-type zinc finger gene family, biotic stress, abiotic stress, transcription factor, tomato

## Abstract

C_2_H_2_-type zinc finger proteins are classic and extensively studied members of the zinc finger family. C_2_H_2_-type zinc finger proteins participate in plant growth, development and stress responses. In this study, 99 C_2_H_2_-type zinc finger protein genes were identified and classified into four groups, and many functionally related *cis*-elements were identified. Differential C_2_H_2_-ZFP gene expression and specific responses were analyzed under drought, cold, salt, and pathogen stresses based on RNA-Seq data. Thirty-two C_2_H_2_ genes were identified in response to multiple stresses. Seven, 3, 5, and 8 genes were specifically expressed under drought, cold, salt, and pathogenic stresses, respectively. Five glycometabolism and sphingolipid-related pathways and the endocytosis pathway were enriched by KEGG analysis. The results of this study represent a foundation for further study of the function of C_2_H_2_-type zinc finger proteins and will provide us with genetic resources for stress tolerance breeding.

## Introduction

Tomato (*Solanum lycopersicum*) is one of the most important vegetable crops of Solanaceae (Mueller et al., 2005). However, the yield and quality of tomato are greatly affected by various biotic and abiotic stresses such as pathogen infection, low temperature, salt, and drought when the plants are exposed to complex environmental conditions. Upon stress perception, transcription factors (TFs) bind to their target genes to regulate their expression and orchestrate biochemical and physiological modifications critical for stress tolerance and the adaptation of plant growth (Hichri et al., 2014).

C_2_H_2_-type zinc finger proteins (C_2_H_2_-ZFPs), which are members of an important TF family, are also called TFIIIA-type ZFPs or classical ZFPs and are widely distributed in eukaryotic genomes (Huang et al., 2005). There are 176, 189, 211, and 109 C_2_H_2_-ZFPs in Arabidopsis, rice, maize, and poplar, respectively (Englbrecht et al., 2004; Agarwal et al., 2007; Liu et al., 2015; Wei et al., 2015). The C_2_H_2_-ZFPs of eukaryotes generally have a specific conserved sequence consisting of 25–30 amino acids: X-X-C-X(1-5)-C-X(12)-H-X(3-6)-H (X: any amino acid; number: the number of amino acids). The two C (Cys) and two H (His) residues in the sequence form a coordination bond with a zinc ion and then form a tetrahedral structure composed of a two-stranded antiparallel β-sheet and an α-helix (Carl et al., 2001). EPF1 (later renamed ZPT2-1) was identified in Petunia as the first plant-specific ZFP. Takatsuji discovered that EPF1 interacts with the promoter region of the 5-enolpyruvylshikimate-3-phosphate synthase gene (EPSPS) and that the expression of EPF1 parallels the expression of EPSPS (Hiroshi et al., 1994). More zinc finger TFs have been subsequently identified in other plants and have been found to play crucial roles in the regulation of development and responses to biotic and abiotic stresses (Sang et al., 2004; Jiang and Pan, 2012).

Many C_2_H_2_-ZFPs genes involved in biotic and abiotic stresses have been studied in detail. GmZF1 and GmZFP3, two soybean C_2_H_2_-ZFPs, positively regulate the cold response and negatively regulate the drought response, respectively. Both of these proteins might be involved in the ABA-dependent pathway during the stress response (Zhang et al., 2016a). *OsMSR15* contains two C_2_H_2_-ZFP motifs, and its expression is strongly upregulated by cold, drought, and heat stresses in different rice tissues at different developmental stages (Zhang et al., 2016b). The expression of a novel ZFP gene, *StZFP1*, cloned from potato, is increased after salt stress as well as after infection by *Phytophthora infestans* and exogenous ABA (Tian et al., 2010). Overexpression of the *CAZFP1* gene, a zinc-finger protein gene isolated from pepper leaves, enhances resistance against infection by the pathogens *Xanthomonas campestris* and *Colletotrichum coccodes* in transgenic Arabidopsis plants (Sang et al., 2004). In tobacco, *ZFT1* functions as a transcription repressor and binds to the EP1S sequence. Overexpression of *ZFT1* renders tobacco plants more tolerant to TMV (Uehara et al., 2005). In this study, C_2_H_2_-ZFP genes are identified from the tomato genome by a bioinformatic analysis method and are analyzed for many aspects, including their phylogenetic relationships, gene structures, conserved protein motifs, chromosomal locations and promoter *cis*-elements. The abiotic and biotic stress response genes of the C_2_H_2_-ZFP family are screened under drought, salt, cold, and pathogen infection stresses based on corresponding RNA-Seq data. The results of the present work will provide us with comprehensive genome-wide knowledge of the tomato C_2_H_2_-ZFP TF family and will also provide us with potential gene resources for biotic and abiotic stress tolerance breeding.

## Materials and Methods

### Identification and Phylogenetic Analysis of the C_2_H_2_-ZFP Gene Family

Tomato genome sequence data were obtained from the Solanaceae Genomics Network (SGN)^1^ (Tomato Genome, 2012). The tomato genome version was GCF_000188115.3_SL2.50. Arabidopsis genes were obtained from the Arabidopsis Information Resource (TAIR^2^). The C_2_H_2_-ZFPs of tomato (SlZFs) were predicted using the HLH hidden Markov model (HMM) profile obtained from Pfam^3^ (PF00096) and analyzed manually using the SMART^4^ database to confirm the presence of C_2_H_2_-ZFP domains (SM000355) [33,34]. Finally, the obtained genes were compared with members of the C_2_H_2_-ZFP gene family in PlnTFDB v3.0^5^ (Riano-Pachon et al., 2007). The ExPaSy site^6^ (Elisabeth et al., 1999) was used to calculate the molecular weights and isoelectric points (pI) of the deduced polypeptides. Multiple sequence alignment was performed using ClustalX 1.83 (Julie et al., 1997). Phylogenetic analyses were performed using the neighbor-joining method in MEGA 5.0 (Tamura et al., 2011) and one unrooted neighbor-joining tree was constructed with 1,000 bootstrap replications.

### Exon/Intron Structure Analysis and Identification of Conserved Motifs

The exon/intron arrangement of the ZFP genes was generated by the GSDS (Gene structure display server)^7^ using both DNA sequences and their corresponding coding sequences (Hu et al., 2015). Additionally, online MEME (Multiple Expectation Maximization for Motif Elicitation)^8^ was performed to search for conserved motifs for each C_2_H_2_-ZFP gene with the following parameters: the maximum number of motifs was set to 15, and the optimum motif width was set to 6 to 50 residues (Bailey et al., 2009). Each structural motif annotation was performed using the Pfam and SMART tools.

### Chromosomal Location

The chromosomal location data of the C_2_H_2_-ZFP genes were obtained from SGN, and a map was generated via MapInspect software^9^. Different colors were used to represent different groups of C_2_H_2_-ZFP genes. Pink, green, blue, and orange represent the I, II, III, and IV groups, respectively. This approach allowed different groups of genes to be more easily distinguished in the distribution of the chromosome.

### Promoter *cis*-Element Analysis

The transcription start site was designated + 1. The promoter sequences (from −2 kb to + 1 bp) of all C_2_H_2_-ZFP genes were obtained from Phytozome and analyzed using the program PlantCARE online^10^. The *cis*-elements were predicted and located.

### Plant Materials and Stress Treatments

Tomato Micro-Tom and CGN18423 (which carries the Cf-19 gene that confers resistance to *Cladosporium fulvum*) were from the Tomato Research Institute of Northeast Agricultural University and were grown in a growth chamber (Conviron, Canada) with a light intensity of 120 μM photons m^–2^ s^–1^ (photoperiod 16 h, day/night temperature 22/18°C). Next, 100 healthy Micro-Tom seedlings were selected for subsequent analyses (10 seedlings used for organ analysis and 90 for drought, salt, cold stress treatments). The tomato seedlings used for drought, salt, and cold stress treatments were transferred to hydroponics and grown for 48 h when they were in the raising period of the four-leaf stage. Then, 30 seedlings were treated with 15% PEG 6000 to simulate drought stress. Young leaves were collected and frozen in liquid nitrogen at 0, 3 and 6 h after drought treatment. Thirty seedlings were transferred to a 5°C growth chamber for cold treatment. Young leaves were collected and frozen in liquid nitrogen at different time points (0, 4, and 12 h) after treatment. Additionally, 30 tomato seedlings were exposed to 200 mM sodium chloride (NaCl). Young leaves were collected and frozen in liquid nitrogen at different time points (0, 2, and 8 h) after treatment. Three biological replicates were performed for each time point. The materials and methods for pathogen stress are shown in Zhao et al. (2019).

### RNA Isolation and cDNA Synthesis

Total RNA was extracted from leaf samples using a plant RNA mini kit (Watson, China) according to the manufacturer’s handbook. The first-strand cDNA was transcribed with the TransScript^®^ One-Step gDNA Removal and cDNA Synthesis SuperMix Kit according to the manufacturer’s instructions.

### Primer Design and qRT-PCR Verification for Different Tissues and Organs

Because of the large number of genes in the gene family and to rule out absolute tissue-specific preferences for subsequent analysis using transcriptome data (all sequencing materials were leaves), 25 genes that distribute in four groups were selected randomly for analysis to determine the tissue – specific general distribution of the family genes. 21 genes were successfully amplified by the primers we designed. The primers were designed by Primer 5.0 software. The primers are shown in Supplementary Table S1. All the primers were checked for primer pair specificity using the NCBI (National Center for Biotechnology Information) tool^11^. The *Solanum lycopersicum* actin (Tom41) gene (GenBank ID: U60480.1) was used as a reference gene. The primer efficiencies are listed in Supplementary Table S1. SYBR^®^ Green Master Mix was used for quantitative PCR in an iQ5 real-time PCR detection system. The 2^–ΔΔCT^ method was used to calculate the gene expression (Livak and Schmittgen, 2001). Three replicates were performed for each abiotic stress treatment, and the standard errors of the three replicates were also calculated.

### cDNA Library Construction and Sequencing

The analysis process of the disease-resistant response transcriptome is described in Zhao et al. (2019). The samples collected under the NaCl, PEG and low temperature stresses mentioned above were used for RNA-Seq analysis, which was completed by the Beijing Genomics Institute (BGI). First, mRNA with a polyA tail was enriched with oligo-dT magnetic beads. Subsequently, rRNA was hybridized with DNA probes, and then, the DNA/RNA hybrid chains were selectively digested by RNaseH. Finally, the DNA probes were digested with DNaseI to purify RNA. After purification, the desired RNA was obtained. The enriched mRNA was broken into short fragments and then reverse transcribed into double stranded cDNA using random hexamer primers. Finally the end of the synthetic double stranded cDNA was smoothed, and the synthetic double stranded cDNA was amplified by PCR. The PCR product was thermally denatured into a single strand, and the single strand DNA was cycled to obtain a single stranded circular DNA library and then sequenced using the Illumina HiSeq platform.

### Reads Mapping, Expression Levels, Gene Annotation, and Enrichment Analysis of C_2_H_2_-Type Zinc Finger TFs

Raw reads obtained from the Illumina HiSeq platform were processed to filter out adapters, shorter reads and low quality reads. The resulting reads (clean reads) were mapped onto the tomato reference genome using HISAT (Kim et al., 2015). To measure the gene expression levels, the total number of fragments per kilobases per million reads (FPKM) of each gene was calculated based on the length of the gene and the counts of reads mapped to the gene. We used getorf (EMBOSS:6.5.7.0) to detect the ORFs of genes. Then, hmmsearch was used to match each ORF to the transcription factor protein domain. Genes were identified according to the transcription factor family characteristics described by PlantTFDB. Then, we classified the C_2_H_2_-type Zinc Finger TFs functionally and used the phyper function in R software for GO and KEGG pathway enrichment analysis.

### Real-Time PCR Validation

The mRNA that remained after cDNA library construction was transcribed using the method mentioned above with the cDNA Synthesis SuperMix Kit. To validate the accuracy of the RNA-Seq assay, 5 genes under abiotic and biotic stresses were selected randomly to confirm their relative expression by qRT-PCR. The primers were designed on NCBI. The primers are shown in Supplementary Table S2. qRT-PCR was applied using the method mentioned above.

## Results

### Identification and Phylogenetic Analyses of C_2_H_2_-ZFP Genes in Tomato

After analysis of the entire genome of the C_2_H_2_-ZFP TF family, 99 C_2_H_2_-ZFPs were identified and named SlZF-1 to SlZF-99. Supplementary Table S3 shows that the molecular masses of the tomato C_2_H_2_-ZFPs range from 11.014 (*SlZF-88*) to 139.814 kDa (*SlZF-4*). The pI values of the predicted proteins varied from 3.5367 (*SlZF-54*) to 10.615 (*SlZF-79*). The lengths of proteins varied from 96 (**SlZF-88**) to 1,253 (*SlZF-4*) amino acids, almost consistent with the molecular masses.

**FIGURE 1:**
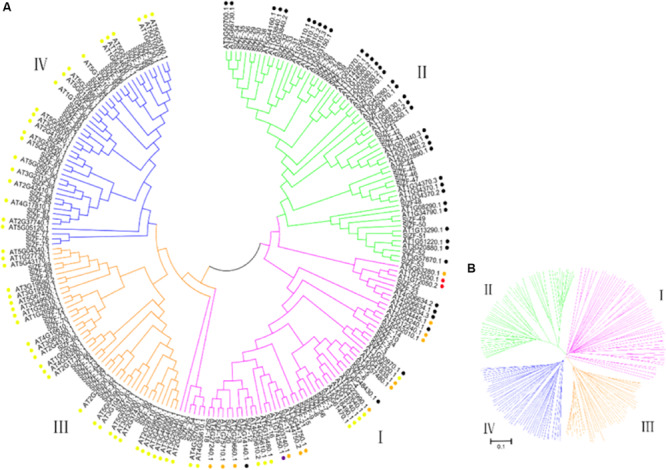
Phylogenetic tree of the C_2_H_2_-ZFPs from tomato and Arabidopsis. **(A)** A phylogenetic tree made from 99 tomato and 116 Arabidopsis ZFPs was constructed using MEGA 5.0 by the neighbor-joining (NJ) method with 1000 bootstrap replicates. The four groups are indicated in four distinct colors. The black spots behind the names of the C_2_H_2_-ZFPs in Arabidopsis represent subset A, the red spots represent subset B, the yellow spots represent subset C1, the orange spots represent subset C2, and the purple spots represent subset C3. **(B)** A phylogenetic tree was constructed to clearly show the four groups of all genes.

The phylogenetic relationship of the C_2_H_2_-ZFPs was determined using the neighbor-joining (NJ) method with 1,000 bootstrap replicates to construct an unrooted phylogenetic tree from alignments of the full-length C_2_H_2_-ZFP sequences of the 99 proteins from tomato and 116 proteins from Arabidopsis (Figure 1A). Figure 1B shows that all the genes were clearly classified into four groups, labeled from I to IV. Group II, which consists of 29 C_2_H_2_-ZFP genes (*SlZF-25* to *SlZF-53*), is the largest group, followed by groups I and IV, containing 26 C_2_H_2_-ZFP genes each (group I: *SlZF-1* to *SlZF-10*, *SlZF-12* to *SlZF-24*, and *SlZF-54* to *SlZF-56*; group IV: *SlZF-11* and *SlZF-75* to *SlZF-99*), and the smallest group is group III, containing 18 C_2_H_2_-ZFP genes (*SlZF-57* to *SlZF-74*). The C_2_H_2_-ZFP family genes of tomato and Arabidopsis are relatively evenly interspersed and distributed among the four groups, indicating that the C_2_H_2_-ZFP genes are closely related in Arabidopsis and tomato.

### Gene Structure and Conserved Motifs of the C_2_H_2_-ZFP Genes in Tomato

**FIGURE 2:**
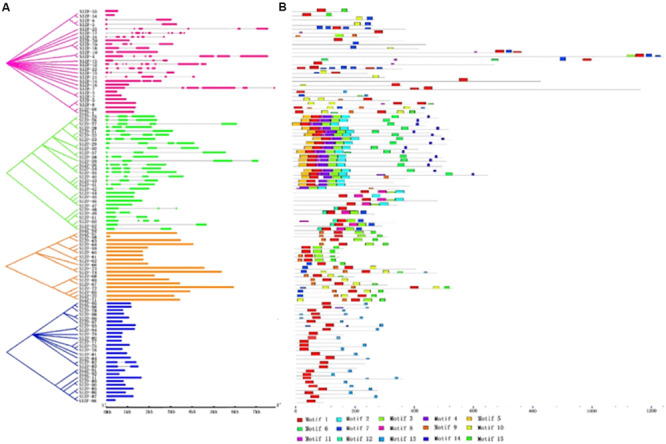
Phylogenetic relations, gene structure and motif compositions of the tomato C_2_H_2_-ZFP genes. The 99 genes were divided into four groups. The four phylogenetic subgroups are marked with different colored backgrounds. **(A)** The exons and introns of each subgroup are represented by particular colored boxes and gray lines, respectively. **(B)** Each colored box represents a motif, and the black lines represent non-conserved sequences.

To examine the structural evolution of the C_2_H_2_-ZFP genes in tomato, the organization of their introns and exons were analyzed using GSDS (Figure 2). Genes in the same group generally had similar numbers of introns. As the schematic structures suggest, the C_2_H_2_-ZFP group III and IV genes (other than *SlZF-82* and *SlZF-83*, which had one intron each) had zero introns. Most of the C_2_H_2_-ZFP genes (69%) in group II had two or three introns. In contrast, the gene structure appeared to be more diverse among the members of group I, with striking differences in their numbers of introns (varying from 0 to 9).

In total, 15 distinct motifs were discovered (Supplementary Table S4). The logos of the 15 motifs are listed in Supplementary Table S5, and the motif distribution in the genes is shown in Figure 2. Most motifs in the same group were distributed similarly. In group II, the linked motifs 5, 1, 11, 4, 3, 2, 6, and 14 and the linked motifs 12, 1, 8, 3, and 7 were exhibited by the groups SlZF-25 to SlZF-39 and SlZF-49 to SlZF-53, respectively. Additionally, all of the C_2_H_2_-ZFPs in group III (16/18) except *SlZF-69* and *SlZF-73* had the linked motifs 1 and 15; those two exceptions both had the linked motifs 9, 1, and 10. Nearly half of the C_2_H_2_-ZFPs in group III (7/18) had the linked motifs 9, 1, 3, and 15. Most of the C_2_H_2_-ZFPs in group IV (21/26) had the linked motifs 1 and 13.

### Chromosomal Locations of Tomato C_2_H_2_-ZFP Genes

**FIGURE 3:**
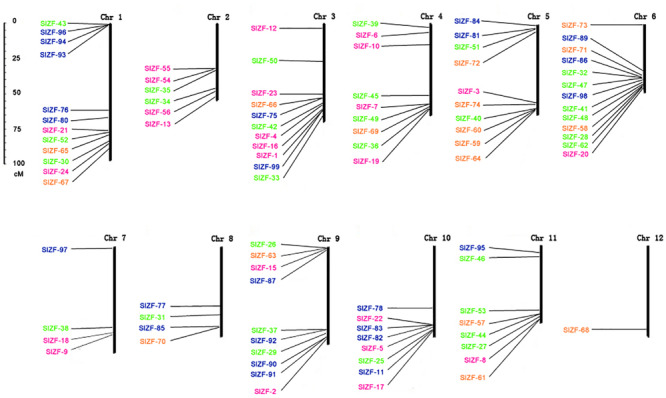
Chromosomal locations of the tomato C_2_H_2_-ZFP genes. Ninety-six out of genes of 99 genes were located on a chromosome. The numbers belonging to same group are marked by the same color. The scale represents centimorgans (cM).

To unravel the evolutionary patterns of this C_2_H_2_-ZFP gene family, we analyzed the locations of the 99 tomato C_2_H_2_-ZFP genes, finding that combinations of the 96 were distributed across all 12 chromosomes (Figure 3). The other three genes, *SlZF-14*, *SlZF-79* and *SlZF-88*, were not be mapped due to the lack of complete annotation in SGN (Sol Genomics Network).

### Promoter *cis*-Element Analysis

**FIGURE 4:**
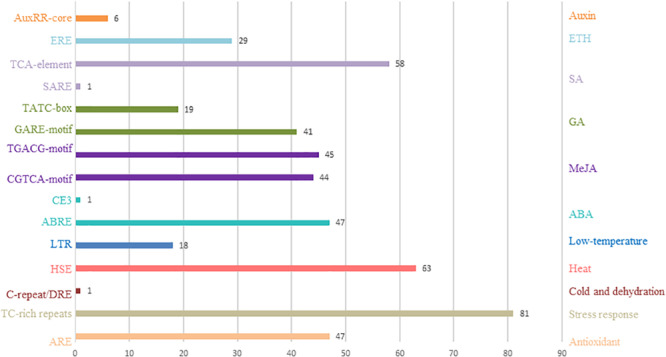
Number of C_2_H_2_-ZFP genes containing various *cis*-acting elements.

Here, analysis of the Plant CARE database revealed multiple *cis*-acting elements in the promoter regions between −1 and 2000 bp upstream of the transcription start sites of all the putative tomato C_2_H_2_-ZFP genes. Figure 4 and Supplementary Table S6 show the presence of multiple *cis*-acting elements related to the phytohormone and environmental stress signal responsiveness of the C_2_H_2_-ZFP family promoters such as the C-repeat/DRE (cold- and dehydration-responsive element), HSE (heat stress-responsive element), LTR (low-temperature-responsive element), ABRE and CE3 (abscisic acid-responsive elements), CGTCA-motif and TGACG (MeJA-responsive elements), GARE-motif and TATC-box (gibberellin-responsive elements), SARE and TCA-element (salicylic acid-responsive elements), and ERE (ethylene-responsive element). The HSE and TCA-element were found in 63 and 58 promoters of the C_2_H_2_-ZFP genes, respectively. Several *cis-*acting elements involved in tissue-specific expression, e.g., an as-2-box (involved in shoot-specific expression), a GCN4_motif (involved in endosperm expression), an HD-Zip 2 (involved in the control of leaf morphology development), and an RY-element (involved in seed-specific regulation), were also found. Several *cis*-elements were unique to individual C_2_H_2_-ZFP genes; for instance, the C-repeat/DRE, CE3, and SARE motifs were present only in SlZF-13, SlZF-45, and SlZF-89, respectively.

### Expression Profiles of the Tomato C_2_H_2_-ZFP Genes in Different Tissues and Organs

**FIGURE 5:**
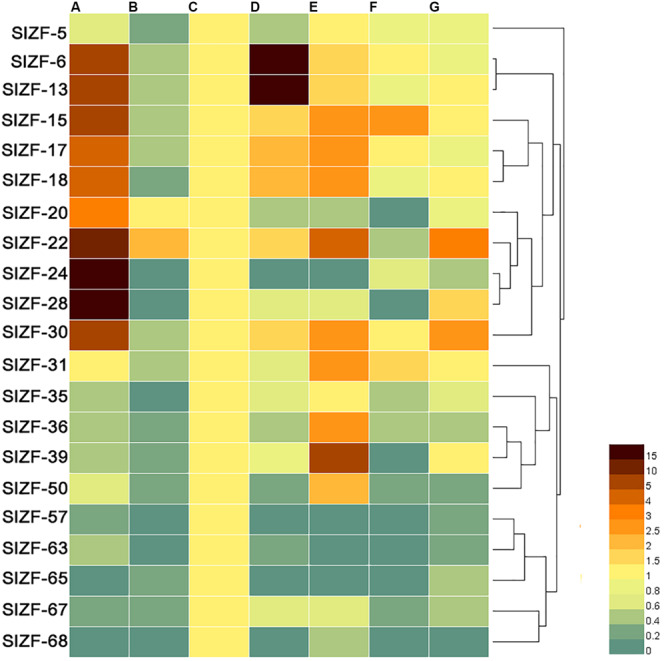
Expression patterns of the C_2_H_2_-ZFP genes in different tomato tissues and organs. **(A)** Whole root; **(B)** young stems; **(C)** mature green leaves; **(D)** flowers at anthesis; **(E)** 20 days post-anthesis fruit; **(F)** breaker stage ripening fruit; **(G)** sepals. All tissues or organs were collected 4 months after sowing. Brown indicates higher expression levels, and bottle green indicates lower expression levels. Genes with similar profiles across arrays are grouped on the right by a hierarchical clustering method.

To further understand the tissue specificity of the C_2_H_2_-ZFP genes, the expression patterns of 21 genes across a variety of tomato tissues, including roots, stems, leaves, flowers, green fruits, mellow fruits and sepals, were analyzed by the qRT-PCR method. As shown in Figure 5, two genes (SlZF-24 and SlZF-28) were significantly highly expressed in root, and two genes (*SlZF-6* and *SlZF-13*) were significantly highly expressed in flower. Furthermore, 10 genes (*SlZF-6, SlZF-13, SlZF-15, SlZF-17, SlZF-18, SlZF-20, SlZF-22, SlZF-24, SlZF-28*, and *SlZF-30*) and eight genes (*SlZF-15, SlZF-17, SlZF-18, SlZF-22, SlZF-30, SlZF-31, SlZF-36*, and *SlZF-39*) were highly expressed in root and green fruit, respectively. *SlZF-22* was expressed in many tissues and organs. Interestingly, all of the 21 genes were expressed similarly in leaves. Genes with similar profiles across arrays were grouped on the right by a hierarchical clustering method.

### C_2_H_2_-ZFP Gene Expression Pattern Analysis Based on RNA-Seq Data

**FIGURE 6:**
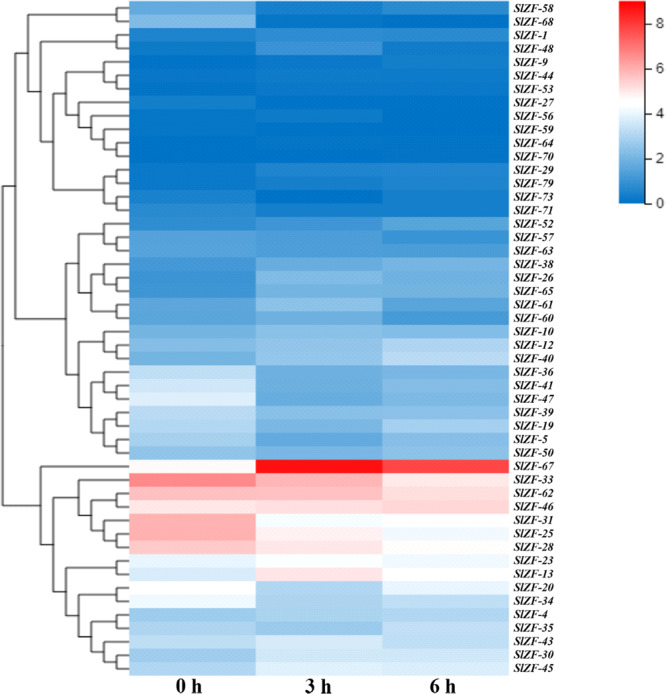
C_2_H_2_-ZFP gene expression under drought stress determined by RNA-Seq. Heatmap of C_2_H_2_-ZFP gene expression under drought stress after 0, 3, and 6 h.

**FIGURE 7:**
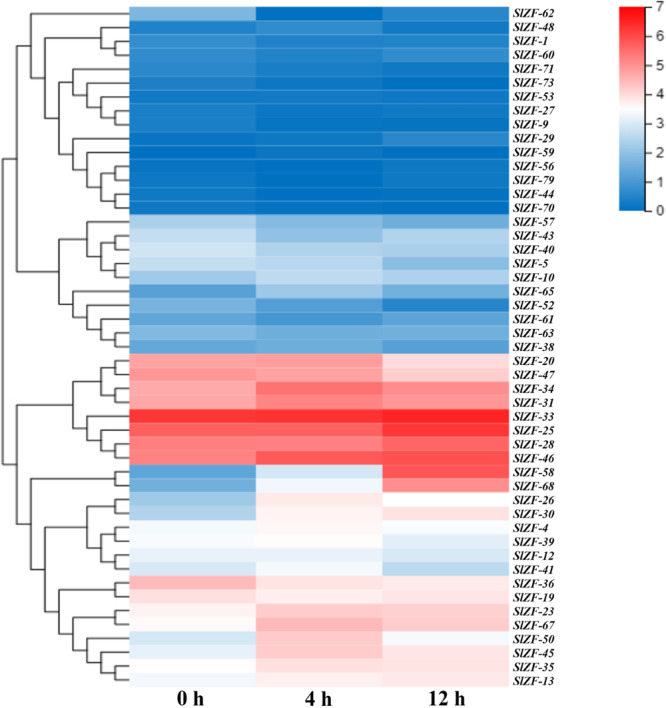
C_2_H_2_-ZFP gene expression under heat stress determined by RNA-Seq. Heatmap of C_2_H_2_-ZFP gene expression under cold stress after 0, 4, and 12 h.

**FIGURE 8:**
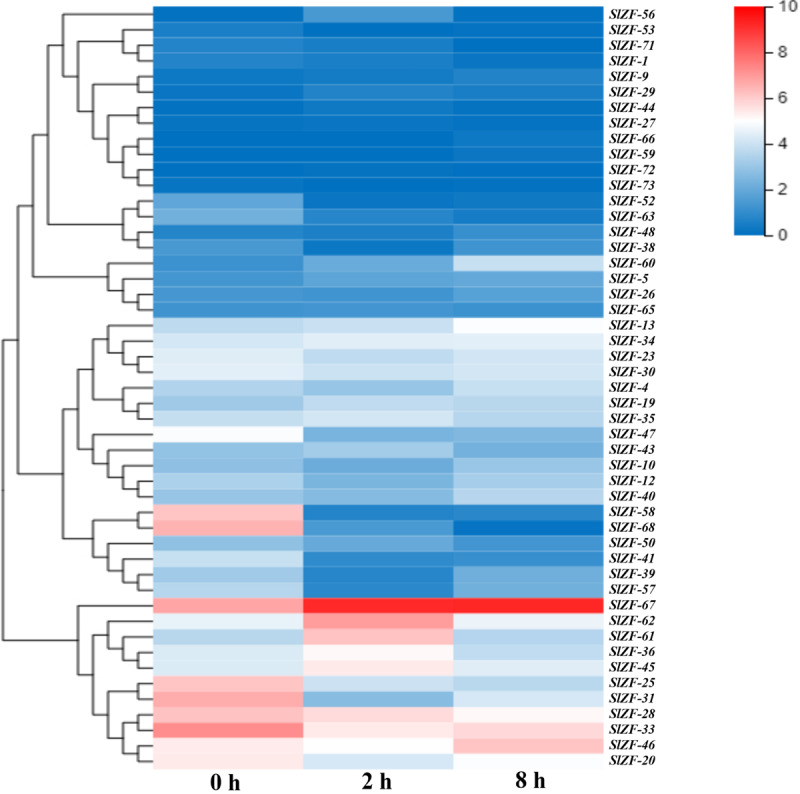
C_2_H_2_-ZFP gene expression under salt stress determined by RNA-Seq. Heatmap of C_2_H_2_-ZFP gene expression under salt stress after 0, 2, and 8 h.

**FIGURE 9:**
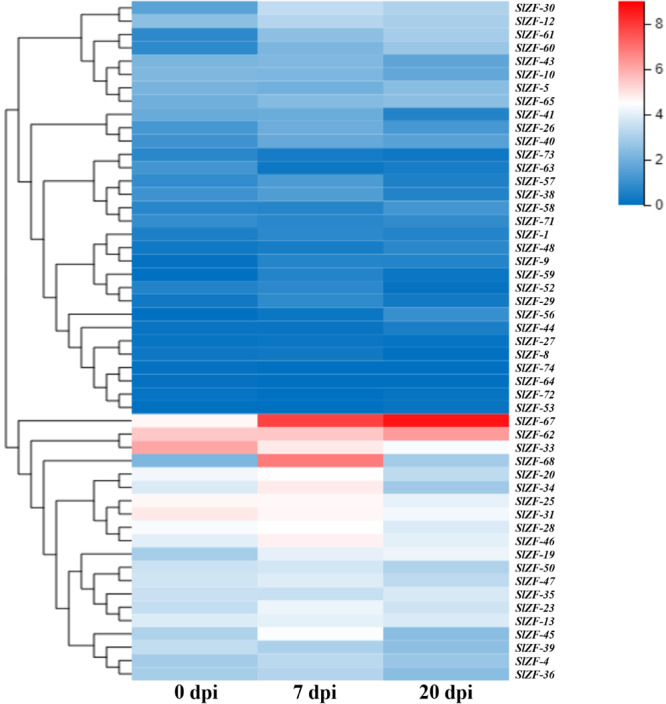
C_2_H_2_-ZFP gene expression under pathogen infection stress. Heatmap of C_2_H_2_-ZFP gene expression under pathogen infection stress at 0, 7, and 20 days.

**FIGURE 10:**
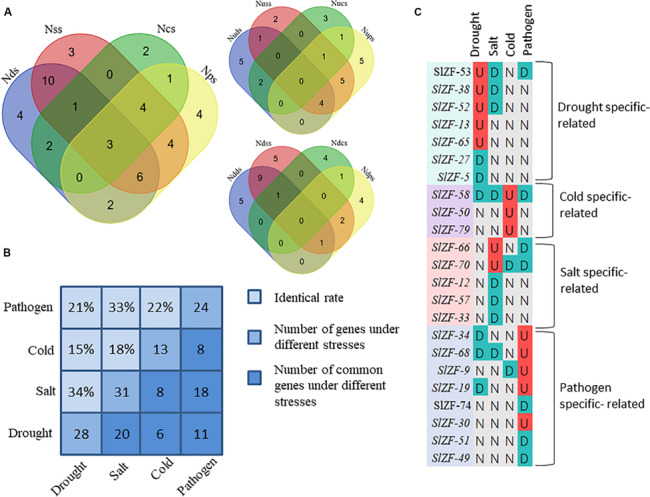
Comparisons of differentially expressed genes and genes with different response patterns under the four stresses. **(A)** Venn diagrams showing the numbers the up- and down-regulated genes in the four comparisons. Nd(s/c/p)s: the number of differentially expressed genes under drought (salt/cold/pathogen) stress; Nud(s/c/p)s: the number of upregulated genes under drought (salt/cold/pathogen) stress; Ndd(s/c/p)s: the number of downregulated genes under drought (salt/cold/pathogen) stress. **(B)** Proportions of the identical DEGs under any two stresses. **(C)** A representation of a gene specifically expressed; “U” means upregulated; “D” means downregulated; “N” means no change.

Four groups of RNA-Seq data, including tomato drought, salt, cold, and pathogen infection response transcriptome data, were used for this analysis. The statistics of the pathogen infection response transcriptome data information are shown in Zhao et al. (2019), and around 6.4 G of data were generated per sample. The details of raw reads, clean reads and read quality of each sample are shown in Supplementary Table S7. Through RNA-Seq data analysis, 50 C_2_H_2_-ZFP genes were screened under drought stress (Figure 6), 49 C_2_H_2_-ZFP genes were screened under cold stress (Figure 7), 50 C_2_H_2_-ZFP genes were screened under salt stress (Figure 8) and 51 genes were screened under pathogen infection (Figure 9). The FPKM of each C_2_H_2_-ZFP genes in three replicates at different time points under the four stresses are shown in Supplementary Tables S8–S11. We screened and identified genes whose absolute value of log_2_ (FPKM of second time point/FPKM of CK0) was greater than 1 as differentially expressed genes (DEGs) and obtained 28 drought-related genes, 31 salt-related genes, 13 cold-related genes and 24 pathogen-related genes. As shown in Figure 10A (a detailed gene list is shown in Supplementary Table S12), a small number of genes was involved in only one stress response, and most genes were involved in more than one stress response. Three genes (*SlZF-58*, *SlZF-61*, *SlZF-5*) participated in all four stress responses. The numbers of upregulated genes and downregulated genes involved in the four stresses were similar. The numbers of upregulated and downregulated genes in the three abiotic stresses were similar, but the number of upregulated genes was significantly higher than the number of downregulated genes under biotic stress (Figure 10A). Further analysis shows (Figure 10B) that the highest proportion of the same response genes was 34% under drought and salt stresses, followed by pathogenic and salt stresses at 33%. Additionally, the lowest proportion of the same response genes was 15% under drought and cold stresses. The statistics of the genes that specifically respond to only one of the four kinds of stresses (including differentially expressed genes under only one stress and genes whose expression patterns were different from those under the other three stresses) were evaluated. The results are shown in Figure 10C. A total of 23 genes were screened out. Seven genes were specifically related to drought stress, five genes were specifically related to salt stress, three genes were specifically related to cold stress, and eight genes were specifically related to pathogen stress.

### GO Enrichment Analysis of the C_2_H_2_-ZFP Genes

**FIGURE 11:**
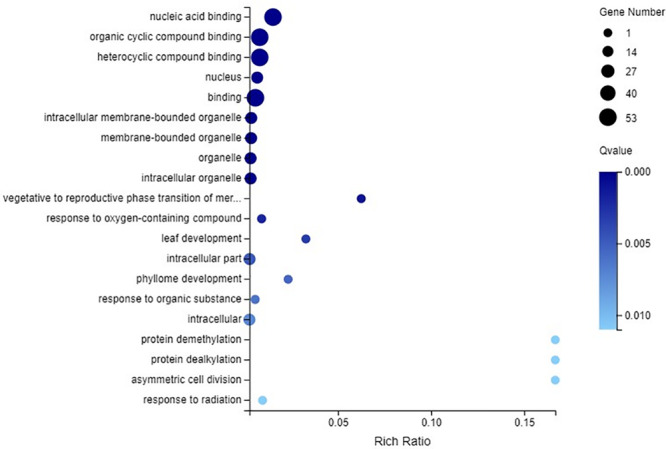
GO enrichment analysis of all the C_2_H_2_-ZFP genes identified through RNA-Seq.

GO enrichment of all the C_2_H_2_-ZFP genes was performed. All 53 C_2_H_2_-ZFP genes identified through transcriptome data were involved in nucleic acid binding, organic cyclic compound binding, heterocyclic compound binding and binding. These four terms all belong to the molecular function category. Twenty C_2_H_2_-ZFP genes were involved in the nucleus, intracellular membrane-bounded organelle, membrane-bounded organelle, organelle and intracellular organelle, which belong to the cellular component category. Additionally, a few C_2_H_2_-ZFP genes were enriched in other GO terms (Figure 11).

### Analysis of the KEGG Metabolic Pathway Enrichment and Expression Patterns in Different Pathways of the C_2_H_2_-ZFP Genes

**FIGURE 12:**
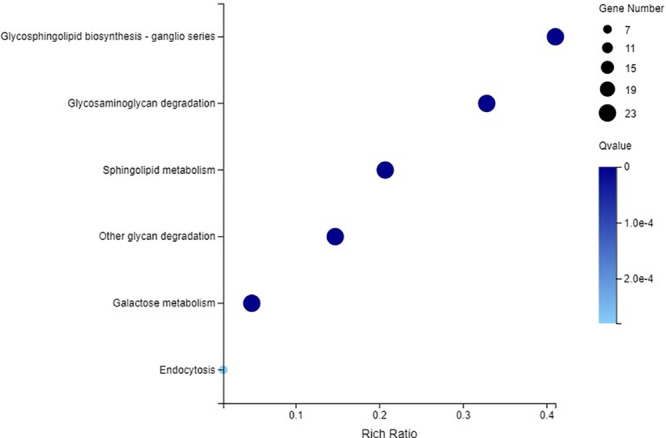
KEGG pathway enrichment analysis of all the C_2_H_2_-ZFP genes identified through RNA-Seq.

**FIGURE 13:**
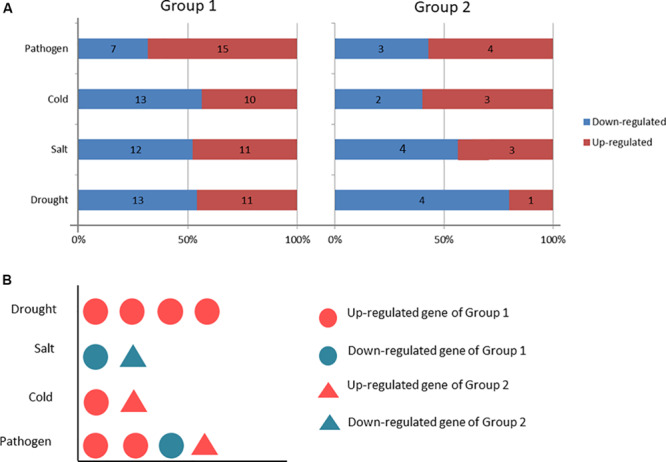
Comparisons of the upregulated and downregulated genes between the two groups. Group1 includes genes from the first five pathways. Group2 contains genes from the sixth pathway. **(A)** Comparison of the upregulated and downregulated gene numbers under different stresses in each group. **(B)** Distribution of the number of genes specifically expressed under the different stresses in the two groups.

The C_2_H_2_-ZFP genes were enriched into 6 KEGG pathways (Q-value ≤ 0.05), including glycosphingolipid biosynthesis, glycosaminoglycan degradation, sphingolipid metabolism, other glycan degradation, galactose metabolism, and endocytosis (Figure 12). Twenty-three C_2_H_2_-ZFP genes were enriched in the first five pathways. Seven C_2_H_2_-ZFP genes were enriched in the endocytosis pathway. Therefore, the genes enriched in these six pathways could be divided into two groups: the genes in the first five pathways were in one group (Group 1), and the genes in the endocytosis pathway were in another group (Group 2). The expression patterns of these genes were statistically analyzed under different stresses, and the results are shown in Figure 13A. The expression patterns of genes in the same group were different under different stresses, and the proportions of upregulated genes and downregulated genes were significantly different. In the first group, there were fewer upregulated genes than downregulated genes under the three abiotic stresses, while the upregulated genes were more numerous than the downregulated genes under biotic stress. In the second group, there were fewer upregulated genes under drought stress and salt stresses, but more upregulated genes under cold stress and pathogen stresses. We further analyzed the distribution of genes that were specifically expressed under each stress in the metabolic pathways. As shown in Figure 13B, 4 genes with specific expression under drought stress belonged to Group 1, and were all upregulated. Two genes were specifically expressed under both salt stress and cold stress; two genes belonging to Group 1 were downregulated under salt stress, and two genes belonging to Group 2 were upregulated under cold stress. Three genes were specifically expressed under pathogen stress in group 1, and 1 gene was specifically expressed under pathogen stress in Group 2, all of which were upregulated except for one gene in Group 1.

### Validation of Gene Expression of Genes via qRT-PCR

**FIGURE 14:**
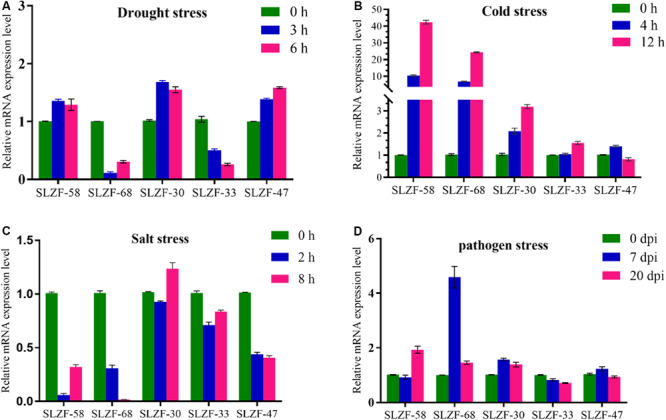
Real-time quantitative PCR validation of five randomly selected genes under the four stresses. **(A)** The expression of the five genes under drought stress measured by qRT-PCR. **(B)** The expression of the five genes under cold stress measured by qRT-PCR. **(C)** The expression of the five genes under salt stress measured by qRT-PCR. **(D)** The expression of the five genes under pathogen stress measured by qRT-PCR. The data were analyzed by three independent repeats, and standard deviations are shown with error bars.

To validate the expression data obtained from RNA-Seq, SlZF-58, SlZF-68, SlZF-30, SlZF-33, and SlZF-47 were chosen for confirmation by qRT-PCR under the four kinds of stresses. The expression levels of the five genes were basically consistent with the results of RNA-Seq (Figure 14). These results indicate that the RNA-Seq results in this study are valid.

## Discussion

### The Evolution of the C_2_H_2_-ZFPs Family Among Different Species Is Conservative

C_2_H_2_-ZFPs are related to plant growth and development, abiotic stress, biotic stress, and other phenomena [14,18,19], showing that C_2_H_2_-ZFPs play an important role in plant life processes. In this study, we identified 99 potential C_2_H_2_-ZFPs in tomato, and the full-length C_2_H_2_-ZFP sequences of these 99 proteins were classified into four groups, labeled from I to IV, through an unrooted phylogenetic tree. We found that the phylogenetic relationship between the C_2_H_2_-ZFPs belonging to set A of the Arabidopsis C_2_H_2_-ZFPs and the group II C_2_H_2_-ZFPs of tomato was close. The phylogenetic relationship between the C_2_H_2_-ZFPs belonging to set C1 of the Arabidopsis C_2_H_2_-ZFPs and groups III and IV C_2_H_2_-ZFPs of tomato was close, though all of the Arabidopsis C_2_H_2_-ZFPs are in group I, which may demonstrate that different plants have conserved different duplicates. Furthermore, the physical parameters of C_2_H_2_-ZFPs in different plants have been shown to be very similar. For example, the molecular masses of the tomato C_2_H_2_-ZFP genes range from 11.014 (SlZF-88) to 139.814 kDa (SlZF-4), the molecular masses of the maize C_2_H_2_-ZFP genes range from 10.535 to 179.433 kDa and the molecular masses of poplar C_2_H_2_-ZFP genes range from 17.626 to 191.195 kDa (Liu et al., 2015; Wei et al., 2015). This similarity suggests that the C_2_H_2_-ZFP family is relatively conserved in evolution. The protein-coding sequences of eukaryotes are interrupted by introns [20]. To further study the distribution of the introns in the genome of tomato, we investigated C_2_H_2_-ZFP TFs and the relationship between tomato C_2_H_2_-ZFP TFs and those of other species. The number of introns within the C_2_H_2_-ZFP groups III and IV genes was either 0 or 1, while gene structure of the numbers within the C_2_H_2_-ZFP groups I and II appeared to be more variable and irregular in terms of intron numbers. These values are very similar to the numbers of Maize and Poplar C_2_H_2_-ZFP introns. Most maize ZmZFP genes in their group C had 0 or 1 intron with the exception of a few members. In contrast, the gene intron–exon structures are more variable in group A and group B, which had more intron numbers. Poplar C_2_H_2_-ZFP genes in their group I and II possessed zero to three introns except for one gene. By contrast, the numbers of introns of genes in groups III and IV are large and irregular (Liu et al., 2015; Wei et al., 2015). Ninety-six of all 99 tomato C_2_H_2_-ZFP genes were unevenly distributed across all 12 chromosomes. Gene duplication plays an important role in genomic expansion (Vision et al., 2000), and the uneven distribution of the locations of the C_2_H_2_-ZFP genes on the chromosomes may be related to the duplication of the tomato genome (Ma et al., 2015). Nevertheless, all four groups of the C_2_H_2_-ZFP gene family were found on chromosomes 1, 3, 5, 6, 9, and 11, suggesting that members of different C_2_H_2_-ZFP gene family groups may interact via a network of proteins.

### C_2_H_2_-ZFPs Gene Families May Have Multiple Functions

Fifteen motifs were found in this study. Most C_2_H_2_-ZFPs in tomato have motif 1, whose best possible match sequence contains the QALGGH amino acid sequence. Many plant ZFPs that are plant specific contain this motif (Agarwal et al., 2007; Liu et al., 2015; Wei et al., 2015). High conservation of the QALGGH sequence is critical for DNA-binding activity (Ken-ichi et al., 1998). In addition, ‘DLELRL’ (motif 13), a hexapeptide motif, has been found in 24 C_2_H_2_-ZFPs. This hexapeptide alone is sufficient to confer the ability to repress transcription to a DNA-binding domain in Arabidopsis (Hiratsu et al., 2004). Another repressor is the ERF-associated amphiphilic repression (EAR) motif, or DLN-box, which has been found in 22 C_2_H_2_-ZFPs. Stress-associated EAR-repressors may control the initiation of stress-activated gene expression under various stresses such as cold, salt, drought, and oxidative stress (Kazan, 2006; Ciftci-Yilmaz et al., 2007; Kielbowicz-Matuk, 2012). Motif 9 is a short, leucine-rich region with the core sequence EXEXXAXCLXXL (L-box) and is thought to play roles in protein–protein interactions or in maintaining folded structures (Kielbowicz-Matuk, 2012). We also discovered many motifs that may be related to DNA-binding or protein–protein interactions.

Most of the C_2_H_2_-ZFPs genes in tomato have abiotic stress-related and stress response *cis-*elements, including C-repeat/DRE, HSE, LTR, and TC-rich repeats. In addition, many C_2_H_2_-ZFPs genes have more than one phytohormone-related *cis-*elements (ABRE, CE3, CGTCA-motif, TGACG, GARE-motif, TATC-box, SARE, TCA-element, and ERE). Phytohormones such as salicylic acid (SA), jasmonic acid (JA), ethylene (ET), and abscisic acid (ABA) play important roles in plant responses to biotic and abiotic stresses and in the regulation of developmental processes (Vision et al., 2000). These findings suggest that C_2_H_2_-ZFPs genes may modulate the biotic and abiotic stress responses directly or indirectly in tomato.

In this study, transcriptome data analysis showed that some of the genes in this family had significant changes in expression under four stresses, indicating that these genes were involved in the stress resistance and disease resistance responses of tomato plants. A C_2_H_2_-ZFP encoded by *OsMSR15* has been previously shown to have positive effects on cold, drought, and heat stress in different tissues of rice at different stages of development (Zhang et al., 2016a). *StZFP1*, which was cloned from potato, can be induced under abiotic stresses, such as salt, drought, and exogenous ABA (Tian et al., 2010). *GmZF1* in soybean regulates the expression of cold-regulated genes in transgenic *Arabidopsis thaliana* (*A. thaliana*) and enhances the anti-cold ability of *A. thaliana* (Yu et al., 2014). GO enrichment results showed that all the C_2_H_2_-ZFP genes screened through RNA-Seq were enriched in the molecular function category, indicating that all the C_2_H_2_-ZFP genes have multiple functions.

In addition, in the analysis of tissue-specific expression, we found that the expression of many genes was obviously tissue-specific. Previous studies have revealed that the C_2_H_2_-ZFP family of TFs have broad expression patterns under normal growth conditions in many organs and tissues. *RABBIT EARS* (*RBE*), a C_2_H_2_-ZFP gene, plays an important role in the development of petals in *A. thaliana* (Takeda et al., 2004). The product of *RBE* inhibits the expression of *TCP4* in *A. thaliana* at the early stage of petal development. The expression of *TCP4* in the flower of rbe-1 mutant plants is significantly higher than that in the flower of wild-type plants, which fully indicates that the expression of *RBE* is related to *TCP4* (Li et al., 2016). In this study, the results showed that *SlZF-24* and *SlZF-28* were significantly highly expressed in root and that *SlZF-6* and *SlZF-13* were preferentially expressed in flower, providing some clues about the functions of these genes, which may be related to root and flower development. Previous studies have revealed that root and flower development are influenced by genes such as *SUPERMAN*, a C_2_H_2_-ZFP in Arabidopsis that is a regulator of floral homeotic genes, and *TaZFP34*, which is a transcriptional repressor in wheat and is involved in modulating the root-to-shoot ratio (Chang et al., 2016).

### Many C_2_H_2_-ZFP Genes of Tomato Respond to Multiple Stresses

Thirty- two genes were involved in differential expression under at least two stresses, while only 13 DEGs were involved in one type of stress, proving that most genes respond to multiple stresses. Previous studies have shown that some of the C_2_H_2_-ZFP genes are indeed associated with multiple stresses. *IbZFP1*, which is a Cys2/His2 zinc finger protein gene from sweet potato, is induced by NaCl and PEG. Overexpression of *IbZFP1* significantly enhances salt and drought tolerance in transgenic Arabidopsis plants (Wang et al., 2015). *ZFP245* is a cold- and drought-responsive gene that encodes a C_2_H_2_-ZFP in rice. Overexpression of *ZFP245* results in enhanced tolerance under drought and cold stresses (Huang et al., 2009). A total of 11 DEGs were involved in the three stresses, and 7 genes had the same expression patterns under all three stresses, indicating that most of the DEGs involved in the three stresses had the same expression pattern. Six DEGs were identified under salt, pathogen and drought stresses, among which 4 genes were all upregulated and 1 gene was downregulated under these three stresses. However, only 1 DEG was the same under cold, drought and salt stresses (*SLZF-73*), and was downregulated under all three stresses. Under the four stresses, the cold-related DEGs were the fewest in number, and the cold-specific response genes were also the fewest in number. Indicating that the C_2_H_2_-ZFP genes have the weakest response to cold stress.

### Differences May Exist Among the Regulation of C_2_H_2_-ZFP Genes Under Different Stresses

KEGG analysis showed that the genes of this family were mainly enriched in six pathways including glycosphingolipid biosynthesis, glycosaminoglycan degradation, sphingolipid metabolism, other glycan degradation, galactose metabolism, and endocytosis. The first five pathways are all related to the function of glycometabolism and sphingolipid, and the endocytosis pathway is related to membrane function, all of these functions are closely related to the resistance of plants to environmental stress (Jae et al., 2008; Ali et al., 2018; Couchoud et al., 2019). In this study, many C_2_H_2_-ZFP genes were differentially expressed in these pathways, suggesting that this family of genes may play a role in stress tolerance regulation through these pathways. In the pathways related to glycometabolism and sphingolipid, the proportion of upregulated C_2_H_2_-ZFP genes under biotic stress was higher than that of the proportion of genes upregulated under abiotic stresses, indicating that the sensitivity of the regulatory points of this family gene in the glycometabolism and sphingolipid pathways to biotic and abiotic stresses is different. In the endocytosis pathway, the proportion of upregulated genes under drought stress was the lowest, which was significantly different from the other three stresses. The proportions of upregulated genes under pathogen stress and cold stress were similar, and the data distribution was very different from that in the glycometabolism and sphingolipid -related pathways, indicating that the regulatory points of C_2_H_2_-ZFP genes in the two pathways are relatively independent. In the analysis of stress-specific response genes, we found that all the drought stress-specific response genes were enriched in the glycometabolism and sphingolipid pathways, and that all of them were upregulated, indicating that the C_2_H_2_-ZFP family has drought-specific response factors and that these response factors regulate the drought stress response only by enhancing processes related to glycometabolism and sphingolipid. There are specific response genes in both types of pathways under other stresses, the most notable of which is salt stress. Additionally, the specific response genes are all downregulated under salt stress. Therefore, it is speculated that under salt, cold and pathogen stresses, although some members of the C_2_H_2_-ZFP family regulate plant’ resistance to environmental stress through the glycometabolism and sphingolipid-related pathways and the endocytosis pathway, the regulation process is more specific under salt stress compared to the processes under cold and pathogen stresses.

## Conclusion

Ninety-nine C_2_H_2_-type zinc finger protein genes were identified and classified into four groups based on phylogenetic analysis. These genes were distributed across all 12 chromosomes and were expressed in different tissues or organs including roots, stems, leaves, flowers and fruits, some of which showed tissue-specific expression patterns. Thirty-two C_2_H_2_-ZFP genes were identified in response to multiple stresses. Seven, 3, 5, and 8 genes were specifically expressed under drought, cold, salt, and pathogen stresses, respectively. KEGG analysis revealed that the genes in the C_2_H_2_-ZFP family mainly exert anti-stress regulation through the glycosphingolipid biosynthesis, glycosaminoglycan degradation, sphingolipid metabolism, other glycan degradation, galactose metabolism, and endocytosis pathways. The results of this study are the foundation for further studies on the function of C_2_H_2_-type zinc finger proteins and will provide a basis for stress tolerance breeding.

## Data Availability Statement

The datasets generated for this study can be found in the NCBI GEO accession GSE148530.

## Author Contributions

XX and TZ designed the study and supervised the project and revised the manuscript. TZ, TW, JZ, TP, and ZW implemented the study and acquired the data. TW and TZ drafted the manuscript. HY and JL helped to drafted the manuscript. All authors read and approved the final manuscript.

## Conflict of Interest

The authors declare that the research was conducted in the absence of any commercial or financial relationships that could be construed as a potential conflict of interest.

## Footnotes

1 http://solgenomics.net

2 http://www.arabidopsis.org/index.jsp

3 http://pfam.xfam.org

4 http://smart.embl-heidelberg.de/

5 http://plntfdb.bio.uni-potsdam.de/v3.0/

6 http://web.expasy.org/protparam/

7 http://gsds.cbi.pku.edu.cn

8 http://meme.nbcr.net/meme/

9 http://mapinspect.software.informer.com

10 http://bioinformatics.psb.ugent.be/webtools/plantcare/html/

11 https://www.ncbi.nlm.nih.gov/tools/primer-blast/
