# Supplementary material for: Genome-Wide Analyses of the Genetic Screening of C2H2-Type Zinc Finger Transcription Factors and Abiotic and Biotic Stress Responses in Tomato (Solanum lycopersicum) Based on RNA-Seq Data
Source: Front Genet. 2020 May 28;11:540. doi: 10.3389/fgene.2020.00540 (PMC7270337; doi:10.3389/fgene.2020.00540)
Supplement: Supplementary file 1 [file Table_1.DOCX]

**Table S1. Primers for the** [**tissue**](javascript:;) [**specificity**](javascript:;) **expression patterns of 21 C2H2-ZFP genes**

| Gene Name | Forward primer | Reverse primer | Product length |
| --- | --- | --- | --- |
| *SlZF-5* | CCTGGCTGCAGAGAGTTCTT | GGCCCAACTAGAGGATGACG | 210 |
| *SlZF-6* | GACATCAGTGTCCAACGGCT | CGGTGTTCACATGCGATTCC | 126 |
| *SlZF-13* | GAGCTTCTCGCTAAGCAGGT | TCATCTGTGCACCACAACCA | 127 |
| *SlZF-15* | GACAGAAGGTTCAGCGGGAA | TCCTAGGCCCAGATGTGGAA | 252 |
| *SlZF-17* | CTGTACAGCCGGGAGATGAC | TCATCCTCGAATGGGTTGGC | 262 |
| *SlZF-18* | TGCAGAGTAGTTGCAGGTCG | CGTGACGCGAAGTCTCACTA | 234 |
| *SlZF-20* | CGGACGGCAATGAAGTGTTG | CTCAATCTCCTCAGGCACCG | 200 |
| *SlZF-22* | ACTGCCAGTCTAGCTACCGT | GCCTCAACAGTCTCCAGCTT | 283 |
| *SlZF-24* | TGGAAGTAGCCGGGACGATA | GATGAGCCTCCTTTCGCAGA | 225 |
| *SlZF-28* | GGGACTTGGATTGGACCCTG | TGTTATGTGCGGCGATTTGG | 200 |
| *SlZF-30* | AGCCACTGGCCCTGAACTTA | TGTTGCACCCATTTGAGCTG | 208 |
| *SlZF-31* | GATGAAGGATTTGGTCGCGG | AAGAGCAGCAGGTGGTGTAG | 141 |
| *SlZF-35* | TTGGATCAACCGGGCAAGAA | GTCACATGGCAATCCAAGGC | 245 |
| *SlZF-36* | GTTCAGCAACAATGCAGCCA | CATTGCCAATCCAAGGCCAC | 113 |
| *SlZF-39* | AGTGCAACTGCCTCATTGTTC | AAGCATTGATGATACCACTATCTGT | 130 |
| *SlZF-50* | AGCTTTGACGACGATGACGA | GCTAAACAAACTCACCTCCTCG | 101 |
| *SlZF-57* | AGACAACCTCCAACCACGAC | AGGGGCTGCAGAGAAGACTA | 162 |
| *SlZF-63* | GCACTGTGGAAGGTGCTAGT | TGCTCTGTGTCCACCAAGTG | 278 |
| *SlZF-65* | TGATGCTGATGACTCGGTTGT | TGAAATCGAACTCACCGGCT | 178 |
| *SlZF-67* | TCAGGTGTAGCGAGTGTCCT | GTAGACGTCGACGGATTGCT | 124 |
| *SlZF-68* | ATCGTCATGGAGGAGCAACC | CCTGACGTGACGATCACAGA | 245 |
| *actin* | GTCCTCTTCCAGCCATCCAT | ACCACTGAGCACAATGTTACCG | 126 |

**Table S2. Primers used for qRT-PCR verification**

| Gene Name | Forward primer | Reverse primer | Product length |
| --- | --- | --- | --- |
| SlZF-58 | TTTGTGGGGCGGAATTCTC | GGCAGGTTCAGATCAAGCGA | 196 |
| SlZF-68 | GTGGTCACAAAAGGCGACAC | TTTCCACCTCATCTTCGCCC | 271 |
| SlZF-30 | GCCACTGGCCCTGAACTTAT | ACCCATTTGAGCTGCCTGTT | 201 |
| SlZF-33 | TTCCGCCATGGCTATTGGTT | TGTTACTCATGGTCGCACCC | 273 |
| SlZF-47 | CGGGAGCTGGTGCGTATAAT | GAACGGAAGCACCACCAAAC | 211 |

**Table S3. Information of 99 C2H2-ZF genes, including the accession number, location (in which chromosome), length, molecular mass and pI**

| Gene name | Accession number | Chromosome | Length (a.a.) | Molecular masses(KD) | pI |
| --- | --- | --- | --- | --- | --- |
| *SlZF-1* | Solyc03g115450.1.1 | 3 | 446 | 49.6326 | 8.3354 |
| *SlZF-2* | Solyc09g090260.1.1 | 9 | 257 | 27.8813 | 9.3916 |
| *SlZF-3* | Solyc05g050170.1.1 | 5 | 186 | 21.1581 | 6.0749 |
| *SlZF-4* | Solyc03g111590.2.1 | 3 | 1253 | 139.814 | 9.1879 |
| *SlZF-5* | Solyc10g080200.1.1 | 10 | 273 | 30.2711 | 8.1347 |
| *SlZF-6* | Solyc04g015570.2.1 | 4 | 278 | 31.4049 | 8.3814 |
| *SlZF-7* | Solyc04g074250.1.1 | 4 | 1178 | 128.573 | 5.1745 |
| *SlZF-8* | Solyc11g072210.1.1 | 11 | 481 | 54.066 | 6.4085 |
| *SlZF-9* | Solyc07g066160.1.1 | 7 | 332 | 36.7912 | 6.6771 |
| *SlZF-10* | Solyc04g028560.2.1 | 4 | 778 | 87.6414 | 7.406 |
| *SlZF-11* | Solyc10g084910.1.1 | 10 | 361 | 40.8888 | 6.1357 |
| *SlZF-12* | Solyc03g025440.2.1 | 3 | 686 | 76.0424 | 7.6876 |
| *SlZF-13* | Solyc02g094170.2.1 | 2 | 255 | 28.8025 | 9.0085 |
| *SlZF-14* | Solyc00g110070.1.1 | - | 840 | 91.7135 | 8.3237 |
| *SlZF-15* | Solyc09g009120.2.1 | 9 | 1044 | 112.997 | 6.7693 |
| *SlZF-16* | Solyc03g115120.1.1 | 3 | 446 | 49.6326 | 8.3354 |
| *SlZF-17* | Solyc10g085560.1.1 | 10 | 308 | 33.6541 | 4.4883 |
| *SlZF-18* | Solyc07g063970.2.1 | 7 | 428 | 46.5602 | 9.0982 |
| *SlZF-19* | Solyc04g081370.2.1 | 4 | 452 | 49.0146 | 9.7313 |
| *SlZF-20* | Solyc06g076820.1.1 | 6 | 381 | 42.7155 | 8.0962 |
| *SlZF-21* | Solyc01g081630.2.1 | 1 | 314 | 34.5354 | 8.7997 |
| *SlZF-22* | Solyc10g077110.1.1 | 10 | 373 | 42.8528 | 7.8373 |
| *SlZF-23* | Solyc03g083310.2.1 | 3 | 383 | 43.6222 | 6.9941 |
| *SlZF-24* | Solyc01g101250.2.1 | 1 | 294 | 33.905 | 6.2104 |
| *SlZF-25* | Solyc10g084180.1.1 | 10 | 494 | 54.5891 | 9.3196 |
| *SlZF-26* | Solyc09g007550.2.1 | 9 | 460 | 51.3146 | 9.7749 |
| *SlZF-27* | Solyc11g069240.1.1 | 11 | 450 | 50.0873 | 9.6335 |
| *SlZF-28* | Solyc06g075250.2.1 | 6 | 529 | 58.1848 | 9.2519 |
| *SlZF-29* | Solyc09g074780.2.1 | 9 | 521 | 57.5351 | 9.1016 |
| *SlZF-30* | Solyc01g099340.2.1 | 1 | 425 | 47.4497 | 8.8514 |
| *SlZF-31* | Solyc08g063040.2.1 | 8 | 468 | 51.8148 | 9.1969 |
| *SlZF-32* | Solyc06g062670.2.1 | 6 | 504 | 56.2951 | 8.5913 |
| *SlZF-33* | Solyc03g121660.2.1 | 3 | 543 | 59.2351 | 8.8675 |
| *SlZF-34* | Solyc02g085580.2.1 | 2 | 528 | 54.9489 | 8.6516 |
| *SlZF-35* | Solyc02g062940.2.1 | 2 | 657 | 68.0076 | 7.9918 |
| *SlZF-36* | Solyc04g080130.2.1 | 4 | 502 | 54.293 | 8.1004 |
| *SlZF-37* | Solyc09g065670.2.1 | 9 | 471 | 51.5025 | 8.9169 |
| *SlZF-38* | Solyc07g053570.2.1 | 7 | 503 | 55.2808 | 8.9851 |
| *SlZF-39* | Solyc04g008500.2.1 | 4 | 518 | 57.5195 | 9.23 |
| *SlZF-40* | Solyc05g054030.2.1 | 5 | 382 | 43.992 | 8.5223 |
| *SlZF-41* | Solyc06g072360.2.1 | 6 | 393 | 44.8256 | 9.0408 |
| *SlZF-42* | Solyc03g098070.2.1 | 3 | 365 | 40.5905 | 9.0976 |
| *SlZF-43* | Solyc01g005060.2.1 | 1 | 340 | 38.6027 | 8.5445 |
| *SlZF-44* | Solyc11g066420.1.1 | 11 | 385 | 43.3577 | 8.6766 |
| *SlZF-45* | Solyc04g056320.1.1 | 4 | 374 | 41.6482 | 7.1774 |
| *SlZF-46* | Solyc11g017140.1.1 | 11 | 486 | 54.3077 | 6.5721 |
| *SlZF-47* | Solyc06g065440.1.1 | 6 | 352 | 39.6452 | 8.1121 |
| *SlZF-48* | Solyc06g074360.2.1 | 6 | 212 | 24.6118 | 7.0986 |
| *SlZF-49* | Solyc04g074320.1.1 | 4 | 274 | 31.1228 | 8.6659 |
| *SlZF-50* | Solyc03g059270.2.1 | 3 | 239 | 27.4912 | 7.5367 |
| *SlZF-51* | Solyc05g009770.1.1 | 5 | 213 | 22.8483 | 7.6923 |
| *SlZF-52* | Solyc01g087050.2.1 | 1 | 299 | 34.0381 | 8.0926 |
| *SlZF-53* | Solyc11g062060.1.1 | 11 | 262 | 29.8483 | 6.8618 |
| *SlZF-54* | Solyc02g061700.1.1 | 2 | 140 | 15.1315 | 3.5367 |
| *SlZF-55* | Solyc02g061660.1.1 | 2 | 196 | 20.8368 | 3.5743 |
| *SlZF-56* | Solyc02g088670.1.1 | 2 | 461 | 50.9372 | 5.7356 |
| *SlZF-57* | Solyc11g066400.1.1 | 11 | 295 | 31.9866 | 8.0268 |
| *SlZF-58* | Solyc06g074800.1.1 | 6 | 327 | 35.8144 | 6.8349 |
| *SlZF-59* | Solyc05g054660.1.1 | 5 | 168 | 19.1487 | 9.7743 |
| *SlZF-60* | Solyc05g054650.1.1 | 5 | 153 | 17.388 | 9.4923 |
| *SlZF-61* | Solyc11g073060.1.1 | 11 | 155 | 17.3691 | 10.2848 |
| *SlZF-62* | Solyc06g075780.1.1 | 6 | 327 | 35.8144 | 6.8349 |
| *SlZF-63* | Solyc09g008440.1.1 | 9 | 311 | 34.9519 | 8.165 |
| *SlZF-64* | Solyc05g055500.1.1 | 5 | 363 | 40.783 | 7.1839 |
| *SlZF-65* | Solyc01g090840.2.1 | 1 | 324 | 36.1883 | 6.6476 |
| *SlZF-66* | Solyc03g093870.1.1 | 3 | 175 | 19.9391 | 9.3053 |
| *SlZF-67* | Solyc01g107170.2.1 | 1 | 311 | 32.928 | 7.5339 |
| *SlZF-68* | Solyc12g088390.1.1 | 12 | 201 | 21.8876 | 8.4948 |
| *SlZF-69* | Solyc04g077980.1.1 | 4 | 261 | 27.8398 | 8.4949 |
| *SlZF-70* | Solyc08g078590.1.1 | 8 | 282 | 32.1372 | 8.5573 |
| *SlZF-71* | Solyc06g060740.1.1 | 6 | 307 | 34.2254 | 8.0935 |
| *SlZF-72* | Solyc05g012490.1.1 | 5 | 532 | 59.5188 | 6.4581 |
| *SlZF-73* | Solyc06g005180.1.1 | 6 | 409 | 46.3741 | 7.0767 |
| *SlZF-74* | Solyc05g050860.1.1 | 5 | 482 | 53.8055 | 6.8442 |
| *SlZF-75* | Solyc03g095270.1.1 | 3 | 239 | 27.4912 | 7.5367 |
| *SlZF-76* | Solyc01g058130.1.1 | 1 | 164 | 18.8075 | 9.1772 |
| *SlZF-77* | Solyc08g059770.1.1 | 8 | 154 | 17.4448 | 9.6863 |
| *SlZF-78* | Solyc10g049870.1.1 | 10 | 173 | 19.498 | 10.5943 |
| *SlZF-79* | Solyc00g014800.1.1 | - | 164 | 18.548 | 10.615 |
| *SlZF-80* | Solyc01g060420.2.1 | 1 | 155 | 17.7111 | 10.368 |
| *SlZF-81* | Solyc05g009170.1.1 | 5 | 287 | 32.5629 | 7.3386 |
| *SlZF-82* | Solyc10g078990.1.1 | 10 | 233 | 26.5596 | 7.7154 |
| *SlZF-83* | Solyc10g078970.1.1 | 10 | 206 | 23.7762 | 8.6616 |
| *SlZF-84* | Solyc05g006310.1.1 | 5 | 251 | 28.3941 | 8.7608 |
| *SlZF-85* | Solyc08g077000.1.1 | 8 | 279 | 31.0808 | 6.3359 |
| *SlZF-86* | Solyc06g061160.1.1 | 6 | 200 | 22.5367 | 5.6232 |
| *SlZF-87* | Solyc09g011120.1.1 | 9 | 277 | 31.6662 | 7.9442 |
| *SlZF-88* | Solyc00g015730.1.1 | - | 96 | 11.014 | 8.2278 |
| *SlZF-89* | Solyc06g053720.1.1 | 6 | 178 | 20.0159 | 9.4082 |
| *SlZF-90* | Solyc09g089590.1.1 | 9 | 198 | 21.9166 | 8.4697 |
| *SlZF-91* | Solyc09g089600.1.1 | 9 | 192 | 22.1997 | 5.8628 |
| *SlZF-92* | Solyc09g066250.1.1 | 9 | 135 | 15.3022 | 6.7758 |
| *SlZF-93* | Solyc01g005200.1.1 | 1 | 297 | 32.9964 | 8.5994 |
| *SlZF-94* | Solyc01g005190.1.1 | 1 | 288 | 32.2803 | 6.5869 |
| *SlZF-95* | Solyc11g011890.1.1 | 11 | 247 | 27.2184 | 6.3928 |
| *SlZF-96* | Solyc01g005130.2.1 | 1 | 251 | 27.5006 | 6.7193 |
| *SlZF-97* | Solyc07g006880.1.1 | 7 | 164 | 18.1418 | 8.8539 |
| *SlZF-98* | Solyc06g068390.1.1 | 6 | 178 | 20.2758 | 8.936 |
| *SlZF-99* | Solyc03g117070.1.1 | 3 | 230 | 25.2177 | 9.9223 |

**Table S4. Motif sequences of the C2HC2-ZF genes and the distribution of motifs within the genes**

| Gene name | MOTIF NUMBER | | | | | | | | | |
| --- | --- | --- | --- | --- | --- | --- | --- | --- | --- | --- |
| *SlZF-1* | 1 | 10 | 6 | 9 | 1 | 10 | 15 |  |  |  |
| *SlZF-2* | 7 | 15 | 13 |  |  |  |  |  |  |  |
| *SlZF-3* | 1 | 13 |  |  |  |  |  |  |  |  |
| *SlZF-4* | 3 | 1 | 7 | 7 |  |  |  |  |  |  |
| *SlZF-5* | 10 | 7 |  |  |  |  |  |  |  |  |
| *SlZF-6* | 10 | 7 |  |  |  |  |  |  |  |  |
| *SlZF-7* | 1 | 1 |  |  |  |  |  |  |  |  |
| *SlZF-8* | 10 | 10 | 11 | 1 | 1 | 15 |  |  |  |  |
| *SlZF-9* | 10 | 10 | 9 | 1 |  |  |  |  |  |  |
| *SlZF-10* | 11 | 1 | 7 | 1 |  |  |  |  |  |  |
| *SlZF-11* | 1 | 13 | 13 |  |  |  |  |  |  |  |
| *SlZF-12* | 1 | 11 |  |  |  |  |  |  |  |  |
| *SlZF-13* | 7 | 10 | 1 |  |  |  |  |  |  |  |
| *SlZF-14* | 1 |  |  |  |  |  |  |  |  |  |
| *SlZF-15* | 6 | 11 | 6 | 7 | 1 |  |  |  |  |  |
| *SlZF-16* | 10 | 1 |  |  |  |  |  |  |  |  |
| *SlZF-17* | 11 | 1 |  |  |  |  |  |  |  |  |
| *SlZF-18* | 7 | 10 |  |  |  |  |  |  |  |  |
| *SlZF-19* | 7 |  |  |  |  |  |  |  |  |  |
| *SlZF-20* | 1 |  |  |  |  |  |  |  |  |  |
| *SlZF-21* | 10 |  |  |  |  |  |  |  |  |  |
| *SlZF-22* | 1 | 1 | 7 | 7 | 7 | 7 | 1 | 7 |  |  |
| *SlZF-23* | 7 | 7 | 1 | 7 | 7 |  |  |  |  |  |
| *SlZF-24* | 9 | 3 | 9 |  |  |  |  |  |  |  |
| *SlZF-25* | 5 | 1 | 11 | 4 | 3 | 2 | 6 | 14 |  |  |
| *SlZF-26* | 5 | 1 | 11 | 4 | 3 | 2 | 6 | 14 |  |  |
| *SlZF-27* | 5 | 1 | 11 | 4 | 3 | 2 | 6 | 14 |  |  |
| *SlZF-28* | 5 | 1 | 11 | 4 | 3 | 2 | 6 | 14 |  |  |
| *SlZF-29* | 5 | 1 | 11 | 4 | 3 | 2 | 6 | 14 |  |  |
| *SlZF-30* | 5 | 1 | 11 | 4 | 3 | 2 | 6 | 14 |  |  |
| *SlZF-31* | 5 | 1 | 11 | 4 | 3 | 2 | 6 | 14 |  |  |
| *SlZF-32* | 5 | 1 | 11 | 4 | 3 | 2 | 6 | 14 |  |  |
| *SlZF-33* | 5 | 1 | 11 | 4 | 3 | 2 | 6 | 14 |  |  |
| *SlZF-34* | 5 | 1 | 11 | 4 | 3 | 2 | 6 | 14 |  |  |
| *SlZF-35* | 5 | 1 | 11 | 4 | 3 | 2 | 4 | 6 | 14 |  |
| *SlZF-36* | 5 | 1 | 11 | 4 | 3 | 2 | 6 | 14 |  |  |
| *SlZF-37* | 5 | 1 | 11 | 4 | 3 | 2 | 6 | 6 | 14 |  |
| *SlZF-38* | 5 | 1 | 11 | 4 | 3 | 2 | 6 | 14 |  |  |
| *SlZF-39* | 5 | 1 | 11 | 4 | 3 | 2 | 6 | 14 |  |  |
| *SlZF-40* | 5 | 1 | 4 | 3 | 2 | 9 |  |  |  |  |
| *SlZF-41* | 5 | 1 | 11 | 4 | 3 | 2 |  |  |  |  |
| *SlZF-42* | 11 | 4 | 3 | 2 | 9 |  |  |  |  |  |
| *SlZF-43* | 5 | 1 | 4 | 3 | 2 |  |  |  |  |  |
| *SlZF-44* | 10 | 8 | 3 | 2 |  |  |  |  |  |  |
| *SlZF-45* | 1 | 8 | 2 |  |  |  |  |  |  |  |
| *SlZF-46* | 10 | 8 | 3 | 2 |  |  |  |  |  |  |
| *SlZF-47* | 10 | 8 | 3 | 2 |  |  |  |  |  |  |
| *SlZF-48* | 12 | 1 |  |  |  |  |  |  |  |  |
| *SlZF-49* | 12 | 1 | 8 | 3 | 7 |  |  |  |  |  |
| *SlZF-50* | 8 | 12 | 1 | 3 | 7 |  |  |  |  |  |
| *SlZF-51* | 12 | 1 | 8 | 3 | 7 |  |  |  |  |  |
| *SlZF-52* | 12 | 1 | 8 | 3 | 7 |  |  |  |  |  |
| *SlZF-53* | 12 | 1 | 8 | 3 | 7 |  |  |  |  |  |
| *SlZF-54* | 1 | 15 |  |  |  |  |  |  |  |  |
| *SlZF-55* | 1 | 15 |  |  |  |  |  |  |  |  |
| *SlZF-56* | 1 | 9 | 10 | 7 | 9 | 10 | 13 |  |  |  |
| *SlZF-57* | 9 | 1 | 3 | 15 |  |  |  |  |  |  |
| *SlZF-58* | 9 | 1 | 3 | 15 |  |  |  |  |  |  |
| *SlZF-59* | 1 | 3 | 15 |  |  |  |  |  |  |  |
| *SlZF-60* | 1 | 3 | 15 |  |  |  |  |  |  |  |
| *SlZF-61* | 9 | 1 | 3 | 15 |  |  |  |  |  |  |
| *SlZF-62* | 9 | 1 | 3 | 15 |  |  |  |  |  |  |
| *SlZF-63* | 9 | 1 | 3 | 15 |  |  |  |  |  |  |
| *SlZF-64* | 9 | 1 | 3 | 15 |  |  |  |  |  |  |
| *SlZF-65* | 7 | 9 | 1 | 10 | 15 |  |  |  |  |  |
| *SlZF-66* | 7 | 1 | 15 |  |  |  |  |  |  |  |
| *SlZF-67* | 9 | 1 | 10 | 15 |  |  |  |  |  |  |
| *SlZF-68* | 9 | 1 | 10 | 15 |  |  |  |  |  |  |
| *SlZF-69* | 9 | 1 | 10 |  |  |  |  |  |  |  |
| *SlZF-70* | 7 | 9 | 10 | 1 | 15 |  |  |  |  |  |
| *SlZF-71* | 10 | 9 | 1 | 3 | 15 |  |  |  |  |  |
| *SlZF-72* | 7 | 10 | 9 | 1 | 10 | 15 |  |  |  |  |
| *SlZF-73* | 7 | 9 | 1 | 10 |  |  |  |  |  |  |
| *SlZF-74* | 7 | 10 | 9 | 1 | 10 | 15 | 13 |  |  |  |
| *SlZF-75* | 1 | 13 |  |  |  |  |  |  |  |  |
| *SlZF-76* | 1 | 13 |  |  |  |  |  |  |  |  |
| *SlZF-77* | 1 |  |  |  |  |  |  |  |  |  |
| *SlZF-78* | 1 | 13 |  |  |  |  |  |  |  |  |
| *SlZF-79* | 1 |  |  |  |  |  |  |  |  |  |
| *SlZF-80* | 1 | 13 |  |  |  |  |  |  |  |  |
| *SlZF-81* | 1 | 13 |  |  |  |  |  |  |  |  |
| *SlZF-82* | 1 |  |  |  |  |  |  |  |  |  |
| *SlZF-83* | 1 |  |  |  |  |  |  |  |  |  |
| *SlZF-84* | 1 | 13 |  |  |  |  |  |  |  |  |
| *SlZF-85* | 1 | 13 |  |  |  |  |  |  |  |  |
| *SlZF-86* | 1 | 13 |  |  |  |  |  |  |  |  |
| *SlZF-87* | 1 | 13 |  |  |  |  |  |  |  |  |
| *SlZF-88* | 1 |  |  |  |  |  |  |  |  |  |
| *SlZF-89* | 1 | 13 |  |  |  |  |  |  |  |  |
| *SlZF-90* | 1 | 13 |  |  |  |  |  |  |  |  |
| *SlZF-91* | 1 | 13 |  |  |  |  |  |  |  |  |
| *SlZF-92* | 1 | 13 |  |  |  |  |  |  |  |  |
| *SlZF-93* | 7 | 1 | 14 | 13 |  |  |  |  |  |  |
| *SlZF-94* | 1 | 13 |  |  |  |  |  |  |  |  |
| *SlZF-95* | 1 | 13 |  |  |  |  |  |  |  |  |
| *SlZF-96* | 1 | 4 | 13 |  |  |  |  |  |  |  |
| *SlZF-97* | 1 | 13 |  |  |  |  |  |  |  |  |
| *SlZF-98* | 13 | 1 | 13 |  |  |  |  |  |  |  |
| *SlZF-99* | 13 | 1 | 13 |  |  |  |  |  |  |  |

**Table S5 Sequence logos for the 15 identified motifs**

| Motif 1 | Motif 2 | Motif 3 | Motif 4 | Motif 5 |
| --- | --- | --- | --- | --- |
| 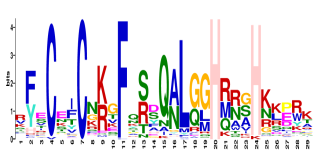 | 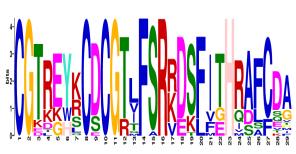 | 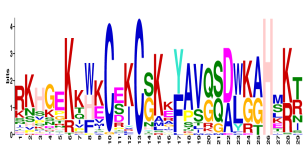 | 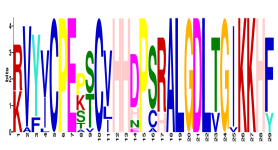 | 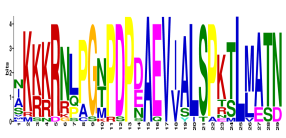 |
| Motif 6 | Motif 7 | Motif 8 | Motif 9 | Motif 10 |
| 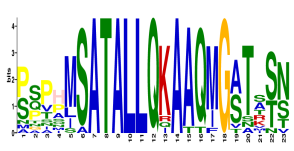 | 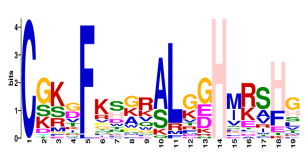 | 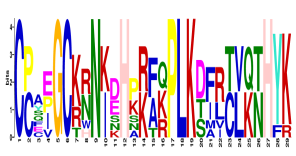 | 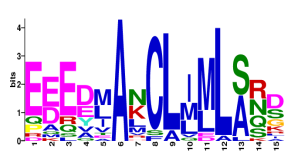 | 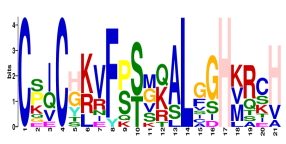 |
| Motif 11 | Motif 12 | Motif 13 | Motif 14 | Motif 15 |
| 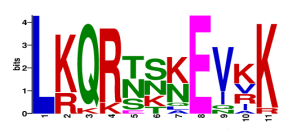 | 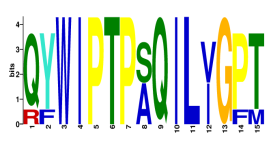 | 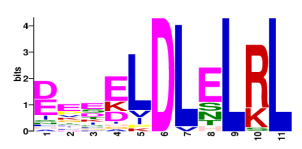 | 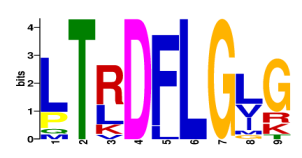 | 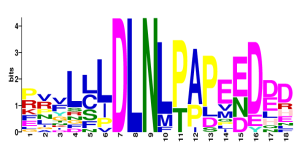 |

**Table S6. Information for some cis-elements**

| Gene name |  |  |  |  |  |  |  |  |  |  |  |  |  |  |  |  |  |  |  |  |
| --- | --- | --- | --- | --- | --- | --- | --- | --- | --- | --- | --- | --- | --- | --- | --- | --- | --- | --- | --- | --- |
|  | ABRE | ARE | AuxRR-core | C-repeat/DRE | CE3 | CGTCA-motif | ERE | GARE-motif | GCN4_motif | HSE | LTR | RY-element | SARE | Skn-1_motif | TATC-box | TC-rich repeats | TCA-element | TGA-element | TGACG-motif | sum |
| *SlZF-1* |  |  |  |  |  | 1 |  |  | 1 | 1 |  | 1 |  | 1 |  |  | 1 |  | 1 | 7 |
| *SlZF-2* |  |  |  |  |  |  | 1 | 1 | 1 | 1 |  |  |  |  | 1 | 1 | 1 |  |  | 7 |
| *SlZF-3* | 1 |  |  |  |  |  |  |  |  | 1 |  |  |  | 1 |  | 1 |  |  |  | 4 |
| *SlZF-4* | 1 |  |  |  |  |  | 1 |  |  |  |  |  |  | 1 |  | 1 | 1 |  |  | 5 |
| *SlZF-5* |  |  |  |  |  |  | 1 |  | 1 |  | 1 |  |  | 1 |  | 1 |  |  |  | 5 |
| *SlZF-6* |  |  |  |  |  | 1 |  |  |  |  |  |  |  | 1 |  | 1 | 1 |  | 1 | 5 |
| *SlZF-7* |  |  |  |  |  | 1 |  |  |  |  |  |  |  | 1 |  | 1 | 1 |  | 1 | 5 |
| *SlZF-8* | 1 |  |  |  |  |  |  | 1 |  | 1 |  |  |  | 1 | 1 | 1 | 1 |  |  | 7 |
| *SlZF-9* | 1 | 1 |  |  |  | 1 | 1 |  |  |  |  |  |  | 1 |  | 1 |  |  | 1 | 7 |
| *SlZF-10* |  |  |  |  |  |  |  | 1 |  |  | 1 |  |  | 1 |  | 1 | 1 |  |  | 5 |
| *SlZF-11* |  | 1 |  |  |  |  |  |  | 1 | 1 | 1 |  |  | 1 |  |  | 1 |  |  | 6 |
| *SlZF-12* |  |  |  |  |  | 1 |  | 1 |  | 1 |  |  |  | 1 |  | 1 | 1 |  | 1 | 7 |
| *SlZF-13* |  | 1 |  | 1 |  | 1 |  | 1 | 1 | 1 |  |  |  | 1 |  | 1 | 1 |  | 1 | 10 |
| *SlZF-14* | 1 |  |  |  |  | 1 |  |  |  |  | 1 |  |  | 1 |  |  |  | 1 | 1 | 6 |
| *SlZF-15* |  | 1 |  |  |  |  | 1 |  |  | 1 |  |  |  | 1 |  | 1 |  |  |  | 5 |
| *SlZF-16* |  | 1 |  |  |  |  | 1 |  |  | 1 |  |  |  | 1 |  | 1 |  |  |  | 5 |
| *SlZF-17* |  |  |  |  |  | 1 |  | 1 |  |  |  | 1 |  | 1 |  | 1 | 1 |  | 1 | 7 |
| *SlZF-18* | 1 | 1 |  |  |  | 1 | 1 |  | 1 |  |  |  |  |  |  | 1 | 1 |  | 1 | 8 |
| *SlZF-19* |  |  |  |  |  | 1 |  |  |  | 1 |  | 1 |  | 1 |  | 1 |  |  | 1 | 6 |
| *SlZF-20* |  |  |  |  |  |  |  | 1 |  | 1 | 1 |  |  | 1 |  | 1 | 1 |  |  | 6 |
| *SlZF-21* |  |  |  |  |  |  | 1 |  | 1 |  |  |  |  |  |  |  |  |  |  | 2 |
| *SlZF-22* |  | 1 |  |  |  | 1 |  |  |  |  |  |  |  | 1 |  | 1 | 1 | 1 | 1 | 7 |
| *SlZF-23* |  | 1 |  |  |  | 1 |  |  |  | 1 | 1 |  |  | 1 | 1 | 1 |  |  | 1 | 8 |
| *SlZF-24* |  |  |  |  |  | 1 |  | 1 | 1 | 1 |  |  |  |  |  | 1 | 1 |  | 1 | 7 |
| *SlZF-25* |  | 1 |  |  |  |  |  | 1 |  | 1 |  |  |  |  |  | 1 |  |  |  | 4 |
| *SlZF-26* |  |  |  |  |  |  |  | 1 |  |  |  |  |  | 1 |  | 1 |  |  |  | 3 |
| *SlZF-27* | 1 |  |  |  |  |  | 1 |  |  | 1 | 1 |  |  | 1 |  | 1 |  |  |  | 6 |
| *SlZF-28* |  | 1 |  |  |  |  | 1 | 1 |  | 1 | 1 |  |  | 1 |  | 1 | 1 |  |  | 8 |
| *SlZF-29* | 1 |  |  |  |  |  | 1 |  |  |  |  |  |  | 1 | 1 | 1 | 1 |  |  | 6 |
| *SlZF-30* |  |  |  |  |  |  |  |  | 1 | 1 |  |  |  | 1 |  | 1 |  |  |  | 4 |
| *SlZF-31* | 1 | 1 |  |  |  |  | 1 |  |  | 1 |  |  |  |  |  | 1 |  |  |  | 5 |
| *SlZF-32* | 1 | 1 |  |  |  |  | 1 |  | 1 | 1 |  |  |  | 1 |  |  | 1 |  |  | 7 |
| *SlZF-33* | 1 |  |  |  |  | 1 | 1 |  | 1 |  |  |  |  | 1 |  | 1 | 1 | 1 | 1 | 9 |
| *SlZF-34* |  |  |  |  |  | 1 |  | 1 |  |  |  |  |  | 1 |  | 1 | 1 |  | 1 | 6 |
| *SlZF-35* | 1 |  | 1 |  |  | 1 |  |  | 1 |  |  |  |  | 1 |  | 1 |  |  | 1 | 7 |
| *SlZF-36* | 1 |  |  |  |  | 1 |  | 1 |  | 1 |  |  |  | 1 |  | 1 |  | 1 | 1 | 8 |
| *SlZF-37* |  |  |  |  |  |  | 1 |  | 1 | 1 |  |  |  | 1 |  | 1 | 1 |  |  | 6 |
| *SlZF-38* |  |  |  |  |  | 1 | 1 | 1 |  | 1 |  |  |  | 1 |  | 1 | 1 |  | 1 | 8 |
| *SlZF-39* |  |  |  |  |  |  |  |  | 1 | 1 | 1 |  |  | 1 | 1 |  |  |  |  | 5 |
| *SlZF-40* | 1 | 1 |  |  |  | 1 |  |  | 1 |  |  |  |  | 1 | 1 | 1 |  | 1 | 1 | 9 |
| *SlZF-41* |  | 1 |  |  |  | 1 |  | 1 | 1 | 1 |  |  |  | 1 |  | 1 |  |  | 1 | 8 |
| *SlZF-42* |  |  |  |  |  | 1 |  |  |  | 1 |  |  |  | 1 |  | 1 | 1 |  | 1 | 6 |
| *SlZF-43* |  |  |  |  |  |  |  |  | 1 | 1 |  |  |  | 1 |  | 1 |  |  |  | 4 |
| *SlZF-44* |  | 1 |  |  |  | 1 |  |  |  | 1 |  |  |  | 1 | 1 | 1 |  | 1 | 1 | 8 |
| *SlZF-45* | 1 |  |  |  | 1 | 1 |  |  |  | 1 |  |  |  | 1 |  |  | 1 |  | 1 | 7 |
| *SlZF-46* |  | 1 | 1 |  |  |  |  | 1 |  | 1 |  |  |  | 1 |  | 1 | 1 | 1 |  | 8 |
| *SlZF-47* |  | 1 |  |  |  |  | 1 | 1 |  | 1 |  |  |  | 1 |  | 1 | 1 |  |  | 7 |
| *SlZF-48* |  | 1 |  |  |  |  |  |  | 1 | 1 |  |  |  | 1 |  | 1 | 1 |  |  | 6 |
| *SlZF-49* | 1 |  |  |  |  | 1 |  |  |  | 1 |  |  |  |  | 1 | 1 |  | 1 | 1 | 7 |
| *SlZF-50* | 1 | 1 |  |  |  | 1 |  |  |  | 1 |  |  |  | 1 |  | 1 | 1 |  | 1 | 8 |
| *SlZF-51* |  | 1 |  |  |  |  | 1 | 1 |  |  |  |  |  | 1 |  | 1 | 1 |  |  | 6 |
| *SlZF-52* |  | 1 |  |  |  |  | 1 |  |  | 1 |  |  |  | 1 |  | 1 |  |  |  | 5 |
| *SlZF-53* |  | 1 |  |  |  |  |  |  | 1 | 1 |  |  |  | 1 |  | 1 |  |  |  | 5 |
| *SlZF-54* |  |  |  |  |  |  |  |  |  | 1 |  |  |  | 1 |  | 1 | 1 | 1 |  | 5 |
| *SlZF-55* |  | 1 |  |  |  | 1 |  | 1 |  | 1 |  |  |  | 1 |  | 1 | 1 | 1 | 1 | 9 |
| *SlZF-56* | 1 | 1 |  |  |  | 1 |  | 1 | 1 |  | 1 |  |  | 1 |  | 1 | 1 |  | 1 | 10 |
| *SlZF-57* |  | 1 |  |  |  |  |  | 1 |  | 1 |  |  |  |  |  |  | 1 | 1 |  | 5 |
| *SlZF-58* |  | 1 |  |  |  | 1 |  | 1 |  | 1 |  |  |  | 1 |  | 1 |  | 1 | 1 | 8 |
| *SlZF-59* | 1 |  |  |  |  |  |  |  |  |  |  |  |  | 1 | 1 | 1 | 1 |  | 1 | 6 |
| *SlZF-60* | 1 | 1 |  |  |  |  |  | 1 |  | 1 |  |  |  | 1 | 1 | 1 | 1 |  |  | 8 |
| *SlZF-61* |  | 1 |  |  |  | 1 |  |  |  | 1 |  |  |  | 1 | 1 | 1 | 1 | 1 |  | 8 |
| *SlZF-62* |  | 1 |  |  |  | 1 |  |  |  | 1 | 1 |  |  | 1 |  |  | 1 |  | 1 | 7 |
| *SlZF-63* |  | 1 | 1 |  |  |  |  | 1 |  | 1 |  |  |  | 1 |  | 1 |  |  |  | 6 |
| *SlZF-64* | 1 | 1 |  |  |  | 1 |  | 1 | 1 | 1 | 1 |  |  | 1 |  | 1 | 1 | 1 | 1 | 12 |
| *SlZF-65* |  | 1 | 1 |  |  |  |  | 1 | 1 | 1 |  |  |  | 1 |  | 1 |  |  |  | 7 |
| *SlZF-66* |  | 1 |  |  |  | 1 |  | 1 |  | 1 |  |  |  | 1 |  |  |  |  | 1 | 6 |
| *SlZF-67* | 1 |  |  |  |  |  |  |  | 1 | 1 |  |  |  | 1 |  | 1 |  |  |  | 5 |
| *SlZF-68* |  |  |  |  |  |  | 1 |  |  |  | 1 |  |  | 1 |  | 1 |  |  |  | 4 |
| *SlZF-69* | 1 |  | 1 |  |  | 1 |  | 1 | 1 | 1 |  |  |  | 1 | 1 | 1 | 1 |  | 1 | 11 |
| *SlZF-70* |  | 1 |  |  |  |  | 1 | 1 |  | 1 | 1 |  |  | 1 |  | 1 | 1 |  |  | 8 |
| *SlZF-71* |  |  |  |  |  |  | 1 | 1 | 1 | 1 |  |  |  | 1 |  | 1 | 1 |  |  | 7 |
| *SlZF-72* |  | 1 |  |  |  |  |  | 1 | 1 | 1 |  | 1 |  | 1 |  | 1 |  |  |  | 7 |
| *SlZF-73* | 1 |  |  |  |  |  | 1 |  | 1 |  |  |  |  | 1 | 1 |  |  |  |  | 5 |
| *SlZF-74* |  | 1 |  |  |  |  |  |  |  |  |  |  |  | 1 |  |  | 1 |  |  | 3 |
| *SlZF-75* | 1 | 1 |  |  |  | 1 | 1 | 1 | 1 |  | 1 |  |  | 1 |  | 1 |  |  | 1 | 10 |
| *SlZF-76* |  | 1 |  |  |  | 1 |  |  |  |  |  |  |  | 1 | 1 | 1 | 1 | 1 | 1 | 8 |
| *SlZF-77* |  | 1 |  |  |  |  |  | 1 |  | 1 |  |  |  | 1 |  | 1 |  |  |  | 5 |
| *SlZF-78* |  | 1 |  |  |  | 1 |  | 1 | 1 | 1 |  |  |  | 1 |  | 1 |  |  | 1 | 8 |
| *SlZF-79* |  |  |  |  |  |  |  |  | 1 |  | 1 |  |  | 1 |  | 1 | 1 | 1 |  | 6 |
| *SlZF-80* |  | 1 |  |  |  | 1 |  |  |  |  |  |  |  | 1 |  | 1 |  |  | 1 | 5 |
| *SlZF-81* |  | 1 |  |  |  | 1 |  | 1 |  | 1 |  | 1 |  | 1 |  | 1 | 1 |  |  | 8 |
| *SlZF-82* |  | 1 | 1 |  |  |  |  | 1 |  | 1 | 1 |  |  | 1 | 1 | 1 |  |  |  | 8 |
| *SlZF-83* |  |  |  |  |  | 1 |  |  | 1 | 1 |  |  |  | 1 |  |  | 1 |  | 1 | 6 |
| *SlZF-84* |  |  |  |  |  |  | 1 | 1 | 1 | 1 |  | 1 |  | 1 |  | 1 | 1 |  |  | 8 |
| *SlZF-85* | 1 |  |  |  |  |  |  |  | 1 | 1 |  |  |  | 1 |  | 1 | 1 | 1 |  | 7 |
| *SlZF-86* |  |  |  |  |  | 1 |  |  |  | 1 |  |  |  | 1 |  |  | 1 |  | 1 | 5 |
| *SlZF-87* |  | 1 |  |  |  | 1 |  |  | 1 |  |  |  |  | 1 | 1 |  | 1 |  | 1 | 7 |
| *SlZF-88* |  |  |  |  |  |  |  |  |  |  |  |  |  |  |  |  |  |  |  | 0 |
| *SlZF-89* |  | 1 |  |  |  | 1 | 1 | 1 |  | 1 |  |  | 1 | 1 |  | 1 | 1 | 1 | 1 | 11 |
| *SlZF-90* |  |  |  |  |  | 1 | 1 |  |  |  |  |  |  | 1 | 1 | 1 | 1 |  | 1 | 7 |
| *SlZF-91* | 1 |  |  |  |  |  |  | 1 |  | 1 |  |  |  | 1 |  | 1 | 1 |  | 1 | 7 |
| *SlZF-92* | 1 | 1 |  |  |  |  |  | 1 | 1 |  |  |  |  |  |  | 1 | 1 |  |  | 6 |
| *SlZF-93* | 1 |  |  |  |  |  |  |  |  | 1 |  |  |  | 1 |  | 1 |  |  | 1 | 5 |
| *SlZF-94* |  |  |  |  |  |  |  |  | 1 |  |  |  |  | 1 |  | 1 | 1 |  |  | 4 |
| *SlZF-95* |  |  |  |  |  |  |  |  | 1 |  |  |  |  | 1 |  | 1 | 1 |  |  | 4 |
| *SlZF-96* |  | 1 |  |  |  | 1 |  | 1 |  |  | 1 |  |  | 1 |  |  | 1 |  | 1 | 7 |
| *SlZF-97* |  |  |  |  |  |  |  |  |  | 1 |  |  |  | 1 |  | 1 | 1 |  |  | 4 |
| *SlZF-98* |  |  |  |  |  |  | 1 |  |  |  |  | 1 |  | 1 | 1 |  |  |  |  | 4 |
| *SlZF-99* |  |  |  |  |  |  | 1 |  |  |  |  | 1 |  | 1 | 1 | 1 |  | 1 |  | 6 |
| sum | 29 | 47 | 6 | 1 | 1 | 44 | 29 | 41 | 37 | 63 | 18 | 8 | 1 | 89 | 19 | 81 | 58 | 19 | 45 |  |

**Table S7 Summary of the sequencing reads after filtering**

| Sample | Total Raw  Read s (Mb) | Total Clean  Read s (Mb) | Total Clean  Base s (Gb) | Clean Reads  Q20 (%) | Clean Reads  Q30 (%) | Clean Reads  Ratio (%) |
| --- | --- | --- | --- | --- | --- | --- |
| drought 0 h 1 | 49.19 | 44.54 | 6.68 | 96.65 | 88.12 | 90.56 |
| drought 0 h 2 | 47.43 | 43.23 | 6.48 | 96.67 | 88.17 | 91.15 |
| drought 0 h 3 | 47.43 | 43.24 | 6.49 | 96.66 | 88.09 | 91.16 |
| drought 3 h 1 | 47.43 | 43.57 | 6.54 | 96.53 | 87.75 | 91.86 |
| drought 3 h 2 | 47.43 | 43.07 | 6.46 | 96.63 | 88.05 | 90.81 |
| drought 3 h 3 | 47.43 | 43.71 | 6.56 | 96.7 | 88.19 | 92.16 |
| drought 6 h 1 | 49.19 | 45.01 | 6.75 | 96.73 | 88.32 | 91.5 |
| drought 6 h 2 | 47.43 | 43.64 | 6.55 | 96.73 | 88.33 | 92 |
| drought 6 h 3 | 47.43 | 43.42 | 6.51 | 96.75 | 88.36 | 91.56 |
| cold 0 h 1 | 52.59 | 42.6 | 6.39 | 96.19 | 87.25 | 81 |
| cold 0 h 2 | 54.34 | 43.56 | 6.53 | 96.11 | 86.97 | 80.17 |
| cold 0 h 3 | 54.34 | 43.28 | 6.49 | 96 | 86.68 | 79.64 |
| cold 4 h 1 | 50.83 | 43.05 | 6.46 | 95.91 | 86.63 | 84.69 |
| cold 4 h 2 | 49.69 | 40.18 | 6.03 | 95.88 | 86.41 | 80.86 |
| cold 4 h 3 | 54.34 | 43.32 | 6.5 | 95.84 | 86.39 | 79.72 |
| cold 12 h 1 | 52.59 | 44.35 | 6.65 | 95.77 | 86.23 | 84.34 |
| cold 12 h 2 | 50.83 | 42.51 | 6.38 | 95.96 | 86.76 | 83.62 |
| cold 12 h 3 | 50.83 | 43.03 | 6.45 | 95.84 | 86.45 | 84.65 |
| salt 0 h 1 | 47.33 | 45.52 | 6.83 | 97.89 | 91.09 | 96.18 |
| salt 0 h 2 | 47.33 | 45.40 | 6.81 | 97.87 | 91.03 | 95.94 |
| salt 0 h 3 | 47.33 | 44.98 | 6.75 | 97.89 | 91.11 | 95.03 |
| salt 2 h 1 | 47.33 | 45.16 | 6.77 | 97.85 | 91.00 | 95.41 |
| salt 2 h 2 | 47.33 | 44.89 | 6.73 | 97.95 | 91.41 | 94.85 |
| salt 2 h 3 | 47.33 | 44.83 | 6.73 | 97.90 | 91.25 | 94.73 |
| salt 8 h 1 | 47.33 | 45.00 | 6.75 | 97.83 | 91.03 | 95.08 |
| salt 8 h 2 | 47.33 | 44.93 | 6.74 | 97.88 | 91.18 | 94.94 |
| salt 8 h 3 | 47.33 | 45.48 | 6.82 | 97.84 | 91.00 | 96.09 |

**Table S8 Expression of C2H2-ZFP genes under drought stress (FPKM).**

| Gene | 0 h (1) | 0 h (2) | 0 h (3) | 3 h (1) | 3 h (2) | 3 h (3) | 6 h (1) | 6 h (2) | 6 h (3) |
| --- | --- | --- | --- | --- | --- | --- | --- | --- | --- |
| SlZF-45 | 7.3 | 8.43 | 8.27 | 13.63 | 13.11 | 13.21 | 12.51 | 13.13 | 13.17 |
| SlZF-67 | 25 | 25.46 | 24.61 | 417.92 | 423.15 | 419.25 | 222.23 | 219.93 | 218.43 |
| SlZF-41 | 10.7 | 10.55 | 11.61 | 2.64 | 2.89 | 3.21 | 3.81 | 3.28 | 3.42 |
| SlZF-66 | 0 | 0 | 0 | 0 | 0.11 | 0 | 0 | 0 | 0 |
| SlZF-1 | 0.64 | 0.35 | 0.76 | 0.3 | 0.1 | 0.15 | 0.29 | 0.15 | 0.35 |
| SlZF-25 | 61.33 | 62.46 | 62.13 | 27.96 | 27.08 | 28.1 | 16.9 | 15.73 | 17.18 |
| SlZF-47 | 13 | 13.66 | 12.21 | 2.46 | 2.32 | 2.66 | 3.46 | 2.82 | 2.93 |
| SlZF-26 | 1.05 | 1.28 | 1.11 | 3.61 | 3.42 | 3.29 | 2.77 | 3.21 | 3.59 |
| SlZF-70 | 0 | 0 | 0 | 0 | 0 | 0 | 0.05 | 0 | 0.05 |
| SlZF-48 | 0.18 | 0.15 | 0.18 | 1.03 | 0.83 | 0.92 | 0.25 | 0.4 | 0.29 |
| SlZF-34 | 16.16 | 17.3 | 17.44 | 6.76 | 6.86 | 7.29 | 8.76 | 9.81 | 9.24 |
| SlZF-73 | 0.39 | 0.28 | 0.2 | 0 | 0.12 | 0.2 | 0.31 | 0.16 | 0.08 |
| SlZF-27 | 0.28 | 0.08 | 0.05 | 0.03 | 0.03 | 0.05 | 0.03 | 0.05 | 0 |
| SlZF-53 | 0.04 | 0.04 | 0.04 | 0.15 | 0 | 0.11 | 0.14 | 0.14 | 0 |
| SlZF-62 | 51.16 | 46.58 | 44.62 | 50.45 | 49.7 | 48.34 | 35.45 | 31.89 | 35.34 |
| SlZF-36 | 8.84 | 8.92 | 8.25 | 2.73 | 3.07 | 2.48 | 3.24 | 2.23 | 2.87 |
| SlZF-39 | 8.03 | 6.53 | 7.99 | 4.07 | 3.19 | 3.29 | 4.26 | 3.39 | 4.68 |
| SlZF-68 | 3.63 | 5.25 | 5.27 | 0.09 | 0 | 0 | 0 | 0.27 | 0.18 |
| SlZF-38 | 1.23 | 1.2 | 1.05 | 2.47 | 3.13 | 3.38 | 3.08 | 2.66 | 3.06 |
| SlZF-63 | 1.79 | 1.69 | 1.85 | 1.54 | 1.53 | 1.29 | 1.44 | 1.17 | 1.23 |
| SlZF-12 | 3.84 | 3.43 | 4.1 | 4.75 | 5.01 | 3.95 | 7.01 | 6.99 | 6.42 |
| SlZF-23 | 15.1 | 14.01 | 14.75 | 19.04 | 19.97 | 18.32 | 16.51 | 17.83 | 15.97 |
| SlZF-5 | 6.22 | 6.73 | 5.77 | 2.27 | 2.63 | 3.14 | 4.01 | 3.96 | 4.81 |
| SlZF-31 | 59.61 | 63.03 | 59.62 | 17.85 | 17.1 | 18.45 | 21.37 | 23.63 | 21.99 |
| SlZF-4 | 5.63 | 5.78 | 5.32 | 6.74 | 7.01 | 6.29 | 7.25 | 7.52 | 7.69 |
| SlZF-50 | 4.61 | 4.38 | 4.17 | 3.23 | 2.27 | 3.61 | 4.35 | 4.41 | 4.92 |
| SlZF-9 | 0.03 | 0.35 | 0.11 | 0.14 | 0.22 | 0 | 0.27 | 0.32 | 0.11 |
| SlZF-20 | 22.28 | 21.59 | 22.63 | 7.27 | 8.24 | 7.85 | 14.86 | 14.61 | 16.2 |
| SlZF-60 | 1.89 | 1.35 | 1.45 | 2.73 | 2.61 | 2.13 | 1.32 | 1.36 | 1.02 |
| SlZF-29 | 0.15 | 0.06 | 0.06 | 0.47 | 0.38 | 0.76 | 0.48 | 0.4 | 0.19 |
| SlZF-28 | 45.77 | 49.64 | 49.07 | 31.74 | 30.3 | 32.44 | 24.38 | 24.67 | 24.31 |
| SlZF-19 | 7.44 | 8.03 | 7.73 | 3.32 | 2.98 | 3.41 | 6.1 | 6.11 | 6.09 |
| SlZF-35 | 7.01 | 7.2 | 7.18 | 5.35 | 5.85 | 5.6 | 8.94 | 9.37 | 8.96 |
| SlZF-43 | 8.65 | 8.96 | 8.63 | 11.68 | 10.76 | 11.43 | 8.69 | 8.68 | 8.17 |
| SlZF-44 | 0.12 | 0.08 | 0.04 | 0.21 | 0.21 | 0.17 | 0.2 | 0.45 | 0.25 |
| SlZF-46 | 31.67 | 31.66 | 33.65 | 34.7 | 33.71 | 31.61 | 38.13 | 37.71 | 37.98 |
| SlZF-33 | 98.95 | 100.03 | 98.84 | 57.69 | 56.47 | 56.7 | 30.8 | 32.45 | 32.64 |
| SlZF-56 | 0.09 | 0.18 | 0.27 | 0.18 | 0.05 | 0.05 | 0 | 0 | 0 |
| SlZF-1 | 0.44 | 0.65 | 0.23 | 0.74 | 0.93 | 0.52 | 0.66 | 0.61 | 0.52 |
| SlZF-64 | 0 | 0 | 0 | 0.07 | 0.04 | 0.04 | 0.04 | 0.04 | 0.04 |
| SlZF-10 | 3.03 | 2.96 | 2.64 | 4.24 | 4.23 | 4.89 | 3.69 | 4.21 | 3.88 |
| SlZF-59 | 0.09 | 0.09 | 0 | 0 | 0 | 0.09 | 0 | 0 | 0 |
| SlZF-40 | 3.02 | 3.16 | 4.14 | 4.86 | 4.52 | 4.7 | 8.11 | 8.41 | 9.37 |
| SlZF-13 | 12.11 | 12.88 | 12.48 | 32.64 | 36.24 | 33.45 | 20.93 | 20.78 | 19.76 |
| SlZF-65 | 1.13 | 0.98 | 1.73 | 3.04 | 3.4 | 3 | 2.94 | 3.16 | 2.99 |
| SlZF-79 | 0.15 | 0.3 | 0.31 | 0.31 | 0.16 | 0.3 | 0.44 | 0.15 | 0.15 |
| SlZF-58 | 2.23 | 2.14 | 2.06 | 0.32 | 0.57 | 0.48 | 0.65 | 0.67 | 0.48 |
| SlZF-56 | 0.09 | 0.18 | 0.27 | 0.18 | 0.05 | 0.05 | 0 | 0 | 0 |
| SlZF-71 | 0.64 | 0.35 | 0.76 | 0.3 | 0.1 | 0.15 | 0.29 | 0.15 | 0.35 |
| SlZF-52 | 0.75 | 0.77 | 0.82 | 1.12 | 1.49 | 1.12 | 1.83 | 1.49 | 1.55 |
| SlZF-57 | 1.75 | 1.41 | 1.46 | 1.51 | 0.9 | 0.93 | 0.99 | 1.26 | 1.17 |
| SlZF-61 | 2.04 | 1.14 | 1.73 | 4.33 | 3.62 | 4.42 | 1.85 | 1.43 | 2.11 |
| SlZF-30 | 5.73 | 6.4 | 5.97 | 11 | 10.61 | 9.22 | 10.74 | 10.59 | 11.71 |

**Table S9 Expression of C2H2-ZFP genes under cold stress (FPKM).**

| Gene | 0 h (1) | 0 h (2) | 0 h (3) | 4 h (1) | 4 h (2) | 4 h (3) | 12 h (1) | 12 h (2) | 12 h (3) |
| --- | --- | --- | --- | --- | --- | --- | --- | --- | --- |
| SlZF-45 | 14.15 | 7.97 | 9.09 | 16.24 | 17.47 | 9.5 | 14.4 | 13.46 | 10.33 |
| SlZF-67 | 37.13 | 10.87 | 16.8 | 26.77 | 20.93 | 10.09 | 19.76 | 17.45 | 16.13 |
| SlZF-41 | 12.29 | 6.82 | 10.51 | 9.2 | 9.21 | 14.36 | 4.15 | 4.99 | 6.79 |
| SlZF-1 | 0.31 | 0.5 | 0.46 | 0.47 | 0.27 | 0.76 | 0.4 | 0.16 | 0.31 |
| SlZF-72 | 0 | 0 | 0 | 0.09 | 0 | 0 | 0.03 | 0 | 0 |
| SlZF-30 | 15.22 | 4.38 | 9.55 | 12.33 | 11.7 | 6.82 | 14.02 | 13.72 | 9.89 |
| SlZF-25 | 40.29 | 50.82 | 23.25 | 90.94 | 50.08 | 65.79 | 64.06 | 75.27 | 56.99 |
| SlZF-47 | 31.95 | 29.54 | 33.19 | 22.95 | 26.32 | 22.55 | 16.75 | 16.79 | 21.03 |
| SlZF-26 | 3.68 | 3.49 | 4.06 | 15.59 | 12.77 | 8.86 | 11.68 | 10.24 | 10.42 |
| SlZF-70 | 0.05 | 0.15 | 0.05 | 0.05 | 0.05 | 0 | 0.15 | 0 | 0.05 |
| SlZF-48 | 0.11 | 0.36 | 0 | 0.27 | 0.6 | 0.66 | 1.02 | 0.19 | 0.33 |
| SlZF-34 | 38.21 | 24.4 | 30.72 | 40.87 | 42.18 | 37.36 | 32.81 | 32.49 | 29.04 |
| SlZF-73 | 0.58 | 0.32 | 0.61 | 0.21 | 0.13 | 0.16 | 0.08 | 0 | 0 |
| SlZF-27 | 0.21 | 0.28 | 0.21 | 0.21 | 0.11 | 0.05 | 0.05 | 0.16 | 0.29 |
| SlZF-53 | 0.18 | 0.18 | 0.36 | 0.34 | 0.2 | 0.47 | 0.14 | 0.15 | 0.22 |
| SlZF-62 | 24.23 | 2.28 | 0 | 5.23 | 0 | 1.72 | 2.12 | 0.46 | 0 |
| SlZF-36 | 31.56 | 20.64 | 16.36 | 14.2 | 13.6 | 18.23 | 13.28 | 12.93 | 13.29 |
| SlZF-39 | 9.18 | 9.62 | 8.22 | 6.1 | 10.61 | 7.83 | 7.66 | 7.61 | 9.88 |
| SlZF-68 | 7.47 | 1.93 | 5.99 | 7.43 | 8.96 | 10.31 | 30.72 | 32 | 17.53 |
| SlZF-38 | 1.54 | 1.58 | 1.61 | 0.73 | 1.84 | 0.99 | 1.04 | 1.26 | 1.88 |
| SlZF-63 | 1.15 | 2.43 | 1.03 | 1.75 | 1.84 | 2.32 | 2.28 | 1.93 | 2.08 |
| SlZF-12 | 10.55 | 8.14 | 10.07 | 7.92 | 8.14 | 7.11 | 7.88 | 6.76 | 6.66 |
| SlZF-23 | 19.14 | 11.81 | 16.04 | 20.05 | 17.31 | 14.11 | 12.38 | 16.69 | 11.88 |
| SlZF-5 | 5.27 | 5.46 | 3.82 | 4.63 | 4.77 | 4.05 | 3.31 | 2.72 | 3.34 |
| SlZF-31 | 61.28 | 25.31 | 53.59 | 21.7 | 35.17 | 28.38 | 27.9 | 29.51 | 50.31 |
| SlZF-4 | 10.23 | 9.23 | 11.09 | 5.65 | 11.24 | 10.47 | 10.12 | 9.71 | 10.54 |
| SlZF-50 | 5.92 | 6.6 | 6.36 | 12.26 | 17.77 | 16.45 | 5.85 | 9.42 | 15.14 |
| SlZF-9 | 0.25 | 0.28 | 0.21 | 0.04 | 0.08 | 0.04 | 0.17 | 0.07 | 0.25 |
| SlZF-20 | 25.37 | 26.1 | 33.48 | 25.71 | 27.8 | 25.11 | 13.81 | 14.96 | 17.83 |
| SlZF-60 | 1.3 | 0.59 | 0.68 | 0.7 | 0.37 | 0.51 | 0.34 | 0.62 | 0.09 |
| SlZF-29 | 0.35 | 0.09 | 0.16 | 0.19 | 0.14 | 0.13 | 0.22 | 0.49 | 0 |
| SlZF-28 | 39.87 | 37.4 | 32.75 | 39.59 | 37.02 | 35.52 | 43.83 | 47.78 | 47.38 |
| SlZF-19 | 8.89 | 14.23 | 13.13 | 7.69 | 12.29 | 12.1 | 16.87 | 13.31 | 19.34 |
| SlZF-61 | 1.66 | 1.51 | 1.25 | 0.39 | 0.94 | 0.38 | 3.79 | 1.39 | 1.65 |
| SlZF-35 | 11.83 | 10.48 | 12.71 | 11.51 | 14.21 | 11.11 | 14.77 | 13.63 | 12.62 |
| SlZF-43 | 11.24 | 5.48 | 3.66 | 5.91 | 2.97 | 5.34 | 2.35 | 4.38 | 4.26 |
| SlZF-52 | 1.33 | 2.1 | 1.31 | 1.65 | 1.21 | 1.8 | 0.57 | 0.45 | 0.64 |
| SlZF-44 | 0 | 0.16 | 0.16 | 0.04 | 0 | 0.25 | 0.28 | 0.04 | 0.17 |
| SlZF-46 | 83.24 | 35.66 | 54.4 | 65.72 | 53.76 | 46.83 | 50.67 | 58.94 | 43.99 |
| SlZF-33 | 98.39 | 75.87 | 62.86 | 92.67 | 78.31 | 79.92 | 85.81 | 90.36 | 76.56 |
| SlZF-56 | 0.55 | 0.09 | 0.05 | 0 | 0 | 0 | 0.09 | 0.14 | 0.09 |
| SlZF-1 | 0.23 | 0.64 | 0.39 | 0.03 | 0.35 | 0.68 | 0.22 | 0.37 | 0.49 |
| SlZF-57 | 4.21 | 4.12 | 6.69 | 3.72 | 2.59 | 1.91 | 1.64 | 1.88 | 2.42 |
| SlZF-10 | 3.76 | 3.61 | 4.58 | 3.05 | 5.03 | 4.48 | 4.5 | 4.25 | 4.4 |
| SlZF-59 | 0.09 | 0 | 0 | 0.09 | 0.1 | 0 | 0.09 | 0 | 0 |
| SlZF-40 | 4.26 | 6.23 | 4.78 | 4.74 | 4.3 | 4.97 | 4.63 | 4.04 | 4.56 |
| SlZF-13 | 12.82 | 9.04 | 12.76 | 14.04 | 12.18 | 10.61 | 11.96 | 13.11 | 10.29 |
| SlZF-65 | 1.52 | 1.29 | 1.31 | 2.36 | 3.44 | 1.5 | 1.85 | 2 | 2.08 |
| SlZF-79 | 0.16 | 0.15 | 0 | 0.62 | 0 | 0.15 | 0 | 0.16 | 0 |
| SlZF-58 | 1.41 | 1.48 | 4.38 | 15.85 | 6.57 | 12.62 | 39.75 | 56.25 | 49.33 |
| SlZF-71 | 0.31 | 0.5 | 0.46 | 0.47 | 0.27 | 0.76 | 0.4 | 0.16 | 0.31 |

**Table S10 Expression of C2H2-ZFP genes under salt stress (FPKM).**

| Gene | 0 h (1) | 0 h (2) | 0 h (3) | 2 h (1) | 2 h (2) | 2 h (3) | 8 h (1) | 8 h (2) | 8 h (3) |
| --- | --- | --- | --- | --- | --- | --- | --- | --- | --- |
| SlZF-45 | 20.96 | 18.97 | 18.49 | 43.39 | 36.87 | 41.78 | 20.35 | 22.22 | 19.92 |
| SlZF-67 | 121.17 | 127.27 | 108.29 | 668.62 | 577.77 | 584.45 | 430.76 | 703.81 | 613.31 |
| SlZF-41 | 8.93 | 16.6 | 13.84 | 0.82 | 0.54 | 0.93 | 2.31 | 1.62 | 1.1 |
| SlZF-66 | 0 | 0 | 0 | 0.21 | 0 | 0 | 0.2 | 0.2 | 0.2 |
| SlZF-1 | 0.49 | 1.14 | 0.63 | 0 | 0.05 | 0.39 | 0.24 | 0.19 | 0 |
| SlZF-72 | 0 | 0 | 0 | 0.08 | 0.05 | 0.08 | 0 | 0.03 | 0.03 |
| SlZF-30 | 20.42 | 11.28 | 20.82 | 17.59 | 14.64 | 14.7 | 14.48 | 15.95 | 16.38 |
| SlZF-25 | 59.7 | 75.1 | 70.3 | 15.53 | 13.97 | 14.97 | 10.11 | 10.02 | 11.41 |
| SlZF-47 | 23.64 | 27.33 | 28.89 | 3.11 | 3.97 | 4.17 | 9.26 | 9.17 | 4.75 |
| SlZF-26 | 1.57 | 1.14 | 1.6 | 1 | 0.97 | 1.39 | 3.74 | 2.89 | 2.24 |
| SlZF-70 | 0 | 0 | 0 | 0.05 | 0.1 | 0 | 0.14 | 0.05 | 0 |
| SlZF-48 | 0.64 | 1.48 | 0.66 | 0.95 | 0.78 | 0.49 | 0.66 | 0.69 | 1.12 |
| SlZF-34 | 15.25 | 15.92 | 16.78 | 19.58 | 16.25 | 20.29 | 39.54 | 30.67 | 20.75 |
| SlZF-73 | 0.04 | 0.08 | 0.12 | 0 | 0 | 0 | 0.04 | 0 | 0.04 |
| SlZF-27 | 0.1 | 0.31 | 0.1 | 0.1 | 0.02 | 0.15 | 0 | 0.02 | 0.07 |
| SlZF-53 | 0.35 | 0.88 | 0.45 | 0 | 0 | 0 | 0.14 | 0.14 | 0.07 |
| SlZF-62 | 17.25 | 36.7 | 22.13 | 99.33 | 129.73 | 121.2 | 17.51 | 11.26 | 23.6 |
| SlZF-36 | 18.06 | 18.23 | 18.56 | 39.75 | 35.74 | 34.23 | 14.9 | 15.14 | 12.69 |
| SlZF-39 | 8.77 | 6.83 | 7.82 | 0.27 | 0.3 | 0.7 | 6.44 | 4.12 | 3.57 |
| SlZF-68 | 101.89 | 67.56 | 90.44 | 0.79 | 0.97 | 1.7 | 0.09 | 0.09 | 0.09 |
| SlZF-38 | 0.95 | 0.72 | 1.67 | 0.15 | 0.12 | 0.18 | 1.51 | 1.13 | 1.34 |
| SlZF-63 | 3.01 | 2.23 | 3.7 | 0.4 | 0.9 | 0.7 | 1.08 | 0.78 | 0.34 |
| SlZF-12 | 9.69 | 6.46 | 9.39 | 4.06 | 3.75 | 4.27 | 9.63 | 8.18 | 8.55 |
| SlZF-23 | 23.51 | 18.15 | 19.81 | 10.94 | 9.75 | 12.38 | 17.42 | 17.28 | 16.19 |
| SlZF-5 | 1.52 | 3.1 | 1.53 | 2.25 | 3.85 | 2.57 | 3.17 | 3.13 | 2.87 |
| SlZF-31 | 92.3 | 120.32 | 97.77 | 3.1 | 3.66 | 5.31 | 18.23 | 14.68 | 17.28 |
| SlZF-4 | 11.3 | 9.13 | 10.16 | 6.49 | 6.25 | 6.76 | 18.4 | 15.76 | 14.04 |
| SlZF-50 | 4.86 | 5.64 | 6.04 | 3.83 | 3.03 | 3.08 | 1.91 | 1.66 | 1.44 |
| SlZF-9 | 0.24 | 0.5 | 0.2 | 0.14 | 0.51 | 0.31 | 0.6 | 0.83 | 0.59 |
| SlZF-20 | 31.89 | 51.45 | 41.75 | 10.3 | 15.82 | 17.28 | 22.71 | 21.88 | 29.16 |
| SlZF-60 | 0.17 | 1.14 | 1.25 | 2.01 | 3.44 | 3.19 | 10.17 | 6.62 | 13.7 |
| SlZF-29 | 0.09 | 0.29 | 0.15 | 0.45 | 0.42 | 0.6 | 0.76 | 0.06 | 0.4 |
| SlZF-28 | 65.37 | 68.6 | 72.24 | 57.71 | 54.64 | 51.49 | 45.79 | 45.79 | 34.26 |
| SlZF-19 | 8.14 | 5.87 | 7.72 | 10.51 | 9.4 | 12.51 | 17.97 | 16.37 | 11.11 |
| SlZF-61 | 12.53 | 12.19 | 11.2 | 53.19 | 47.13 | 71.54 | 8.53 | 8.53 | 10.62 |
| SlZF-35 | 12.94 | 11.81 | 13.41 | 16.49 | 13.73 | 16.63 | 18.03 | 15.15 | 10.73 |
| SlZF-43 | 5.79 | 12.2 | 6.23 | 6.88 | 6.56 | 8.19 | 5.69 | 5.28 | 3.82 |
| SlZF-52 | 3.21 | 3.24 | 2.69 | 0.19 | 0.09 | 0.14 | 0.69 | 0.46 | 0.23 |
| SlZF-44 | 0.2 | 0.15 | 0.04 | 0.51 | 0.16 | 0.24 | 0.27 | 0.12 | 0.08 |
| SlZF-46 | 43.62 | 37.02 | 40.96 | 33.73 | 35.36 | 31.23 | 75.74 | 74.96 | 69.26 |
| SlZF-33 | 140.67 | 165.7 | 148.73 | 39.11 | 34.54 | 42.11 | 48.41 | 51.59 | 52.68 |
| SlZF-56 | 0 | 0.13 | 0.04 | 4.5 | 1.96 | 1.65 | 0.17 | 0.21 | 0.04 |
| SlZF-1 | 0.44 | 0.88 | 0.61 | 0.28 | 0.31 | 0.43 | 0.15 | 0.15 | 0.15 |
| SlZF-64 | 0 | 0 | 0 | 0.04 | 0 | 0 | 0 | 0 | 0 |
| SlZF-57 | 10.93 | 6.42 | 10.82 | 0.19 | 0.28 | 0.8 | 4.49 | 3.75 | 3.74 |
| SlZF-10 | 5.87 | 4.77 | 5.94 | 3.66 | 3.34 | 3.32 | 7.57 | 7.85 | 6.95 |
| SlZF-59 | 0 | 0.09 | 0 | 0 | 0 | 0 | 0 | 0 | 0.17 |
| SlZF-40 | 5.51 | 6.03 | 6.86 | 5.35 | 4.44 | 5.08 | 11.53 | 12.56 | 10.98 |
| SlZF-13 | 12.61 | 13.66 | 12.04 | 13.92 | 14.93 | 14.09 | 24.4 | 27.45 | 29.18 |
| SlZF-65 | 2 | 2.11 | 1.34 | 0.94 | 1.08 | 1.45 | 2.44 | 0.8 | 1.27 |
| SlZF-58 | 79.17 | 19.41 | 69.79 | 0.49 | 0.38 | 0.61 | 1.57 | 1.43 | 0.77 |
| SlZF-71 | 0.49 | 1.14 | 0.63 | 0 | 0.05 | 0.39 | 0.24 | 0.19 | 0 |

**Table S11 Expression of C2H2-ZFP genes under pathogen stress (FPKM).**

| Gene | 0 d (1) | 0 d (2) | 0 d (3) | 7 d (1) | 7 d (2) | 7 d (3) | 20 d (1) | 20 d (2) | 20 d (3) |
| --- | --- | --- | --- | --- | --- | --- | --- | --- | --- |
| SlZF-45 | 7.55 | 5.5 | 3.59 | 20.46 | 21.41 | 15.1 | 4.37 | 4.85 | 9.1 |
| SlZF-67 | 24.21 | 8.14 | 10.73 | 230.41 | 354.09 | 176.47 | 403.92 | 402.1 | 454.07 |
| SlZF-41 | 2.55 | 2.38 | 2.21 | 2.67 | 1.77 | 1.95 | 0.51 | 1.8 | 3.15 |
| SlZF-66 | 0 | 0 | 0.17 | 0 | 0 | 0 | 0 | 0 | 0 |
| SlZF-72 | 0.08 | 0.12 | 0.04 | 0 | 0 | 0.05 | 0.09 | 0.04 | 0.04 |
| SlZF-30 | 1.97 | 1.14 | 1.19 | 9.51 | 10.24 | 8.85 | 7.49 | 2.96 | 5.7 |
| SlZF-25 | 24.36 | 20.57 | 18.22 | 23.6 | 24.15 | 16.35 | 15.59 | 11.96 | 18.18 |
| SlZF-47 | 11.73 | 13.07 | 7.13 | 14.03 | 17.46 | 18.21 | 9.2 | 6.94 | 9.47 |
| SlZF-26 | 1.33 | 1.37 | 0.82 | 2.81 | 1.97 | 1.61 | 1.39 | 1.02 | 1.93 |
| SlZF-48 | 0.21 | 0.27 | 0.11 | 0.33 | 0.35 | 0.42 | 0.67 | 0.11 | 0.44 |
| SlZF-34 | 13.59 | 5.95 | 6.21 | 27.77 | 24.82 | 33.28 | 6 | 5.3 | 9.15 |
| SlZF-73 | 0.65 | 0.41 | 0.19 | 0.3 | 0.64 | 0.07 | 0.19 | 0.12 | 0.18 |
| SlZF-27 | 0.11 | 0.19 | 0.16 | 0.15 | 0.04 | 0.51 | 0.08 | 0.08 | 0.23 |
| SlZF-53 | 0 | 0.05 | 0.06 | 0 | 0 | 0 | 0.11 | 0 | 0.22 |
| SlZF-62 | 43.32 | 11.06 | 8.27 | 44.62 | 65.98 | 51.53 | 76.61 | 109.81 | 85.15 |
| SlZF-36 | 6.73 | 2.64 | 3.77 | 7.91 | 13.13 | 8.34 | 4.35 | 7.09 | 6.04 |
| SlZF-39 | 9.83 | 15.41 | 8.06 | 7.14 | 9.05 | 12.22 | 4.64 | 2.96 | 6.49 |
| SlZF-68 | 3.64 | 0.8 | 2.12 | 114.12 | 164.84 | 136.78 | 6.44 | 3.19 | 6.4 |
| SlZF-38 | 1.12 | 0.81 | 0.84 | 1.65 | 1.36 | 0.76 | 0.52 | 0.47 | 1.26 |
| SlZF-63 | 1.29 | 2.34 | 2.37 | 0.16 | 0.49 | 0.59 | 0.32 | 0.7 | 1.41 |
| SlZF-12 | 4.61 | 5.09 | 5 | 8.08 | 10.13 | 8.56 | 6.76 | 4.43 | 7.06 |
| SlZF-23 | 9.91 | 10.09 | 9.47 | 17.09 | 19.31 | 18.66 | 11.57 | 8.93 | 10.66 |
| SlZF-5 | 3.3 | 4.56 | 2.53 | 2.96 | 2.56 | 3.17 | 4.36 | 2.99 | 4.39 |
| SlZF-31 | 29.03 | 32.4 | 25.34 | 24.05 | 22.76 | 18.42 | 18.52 | 22.9 | 18.7 |
| SlZF-4 | 6.39 | 6.19 | 6.66 | 8.77 | 7.38 | 7.31 | 5.48 | 3.42 | 5.65 |
| SlZF-50 | 11 | 5.04 | 8.63 | 12.15 | 8.81 | 8.64 | 7.56 | 6.84 | 7.66 |
| SlZF-9 | 0.05 | 0.15 | 0.16 | 0.58 | 0.67 | 0.64 | 0.54 | 0.59 | 0.37 |
| SlZF-20 | 17.97 | 9.08 | 11.84 | 21.38 | 19.94 | 17.55 | 9.09 | 11.38 | 16.77 |
| SlZF-60 | 0.77 | 0.13 | 0.67 | 3.56 | 14.39 | 3.71 | 5.59 | 16.59 | 4.49 |
| SlZF-29 | 0.23 | 0 | 0 | 0.79 | 1.28 | 0.51 | 0.24 | 0 | 0.52 |
| SlZF-28 | 20.15 | 16.57 | 18.7 | 21.69 | 19.19 | 14.87 | 13.44 | 12.12 | 10.4 |
| SlZF-19 | 6.73 | 8.64 | 9.1 | 15.37 | 17.81 | 16.97 | 17.21 | 15.88 | 19.51 |
| SlZF-61 | 0.73 | 0.14 | 1.37 | 4.64 | 5.23 | 4.85 | 6.48 | 12.37 | 7.33 |
| SlZF-35 | 10.54 | 11.44 | 10.08 | 10.45 | 11.27 | 10.07 | 12.88 | 8.57 | 10.68 |
| SlZF-43 | 3.46 | 2.04 | 1.84 | 3.76 | 4.29 | 3.08 | 2.14 | 2.55 | 4.38 |
| SlZF-52 | 0.5 | 0.35 | 0.45 | 0.73 | 0.46 | 1.28 | 0.07 | 0.96 | 0.37 |
| SlZF-8 | 0.17 | 0 | 0 | 0.23 | 0.06 | 0 | 0 | 0.06 | 0.06 |
| SlZF-44 | 0.12 | 0.18 | 0 | 0.12 | 0.65 | 0.2 | 0.38 | 0 | 0.19 |
| SlZF-46 | 15.05 | 14.21 | 12.09 | 25.98 | 28.97 | 21.41 | 15.15 | 14.91 | 17.06 |
| SlZF-33 | 67.16 | 32.57 | 37.25 | 28.63 | 41.9 | 25.98 | 20.15 | 26.44 | 24.92 |
| SlZF-56 | 0 | 0 | 0.07 | 0.14 | 0.65 | 0.15 | 0.97 | 0.82 | 2.94 |
| SlZF-74 | 0.05 | 0 | 0 | 0 | 0 | 0 | 0 | 0 | 0 |
| SlZF-64 | 0.05 | 0.11 | 0.06 | 0 | 0.24 | 0 | 0 | 0.23 | 0.11 |
| SlZF-57 | 0.86 | 2.14 | 1.72 | 1.54 | 2.57 | 1.68 | 0.45 | 1.26 | 0.89 |
| SlZF-10 | 3.74 | 2.9 | 2.52 | 3.59 | 4.14 | 3.36 | 2.32 | 1.85 | 2.57 |
| SlZF-59 | 0 | 0 | 0 | 0.55 | 1.02 | 0.75 | 0.14 | 0 | 0 |
| SlZF-40 | 1.11 | 3.39 | 2.19 | 2.48 | 3.12 | 2.13 | 1.91 | 1.01 | 3.38 |
| SlZF-13 | 13.83 | 10.77 | 10.67 | 15.56 | 19.59 | 17.59 | 13.4 | 9.7 | 9.4 |
| SlZF-65 | 2.95 | 3.08 | 3.3 | 4.15 | 6.41 | 6.08 | 4.51 | 2.41 | 6.11 |
| SlZF-58 | 0.64 | 0.81 | 2.06 | 0.59 | 0.31 | 0.72 | 1.27 | 0.42 | 0.48 |
| SlZF-71 | 0.89 | 0.52 | 1.4 | 0.68 | 0.56 | 0.58 | 0.85 | 0.08 | 0.54 |
| SlZF-1 | 0.42 | 1.08 | 0.39 | 0.72 | 0.2 | 0.42 | 0.54 | 0.24 | 0.78 |

**Table S12 Gene details for each part of the Venn diagram**

| **Conditions** | cold drought pathogen salt | cold drought salt | drought pathogen salt | cold pathogen salt | drought salt | cold drought | drought pathogen | pathogen salt | cold pathogen | drought | salt | cold | pathogen |
| --- | --- | --- | --- | --- | --- | --- | --- | --- | --- | --- | --- | --- | --- |
| **Number of genes** | 3 | 1 | 6 | 4 | 10 | 2 | 2 | 4 | 1 | 4 | 3 | 2 | 4 |
| **Elements** | *SlZF-58 SlZF-61*  *SlZF-59* | *SlZF-73* | *SlZF-44 SlZF-53*  *SlZF-67 SlZF-29*  *SlZF-36*  *SlZF-68* | *SlZF-70*  *SlZF-62 SlZF-56*  *SlZF-72* | *SlZF-39*  *SlZF-64 SlZF-20 SlZF-31*  *SlZF-25*  *SlZF-71 SlZF-38*  *SlZF-47 SlZF-52*  *SlZF-41* | *SlZF-26 SlZF-48* | *SlZF-34*  *SlZF-19* | *SlZF-60 SlZF-45 SlZF-66 SlZF-63* | *SlZF-9* | *SlZF-13*  *SlZF-65*  *SlZF-27*  *SlZF-5* | *SlZF-12*  *SlZF-57*  *SlZF-33* | *SlZF-50 SlZF-79* | *SlZF-74*  *SlZF-30*  *SlZF-51 SlZF-49* |
